# Super-Resolution Microscopy Reveals That Stromal Interaction Molecule 1 Trafficking Depends on Microtubule Dynamics

**DOI:** 10.3389/fphys.2021.762387

**Published:** 2021-11-05

**Authors:** Yi-Ting Huang, Ya-Ting Hsu, Yih-Fung Chen, Meng-Ru Shen

**Affiliations:** ^1^Department of Pharmacology, National Cheng Kung University Hospital, College of Medicine, National Cheng Kung University, Tainan, Taiwan; ^2^Institute of Basic Medical Sciences, National Cheng Kung University Hospital, College of Medicine, National Cheng Kung University, Tainan, Taiwan; ^3^Institute of Clinical Medicine, National Cheng Kung University Hospital, College of Medicine, National Cheng Kung University, Tainan, Taiwan; ^4^Division of Hematology, Department of Internal Medicine, National Cheng Kung University Hospital, College of Medicine, National Cheng Kung University, Tainan, Taiwan; ^5^Graduate Institute of Natural Products, College of Pharmacy, Kaohsiung Medical University, Kaohsiung, Taiwan; ^6^Department of Medical Research, Kaohsiung Medical University Hospital, Kaohsiung, Taiwan; ^7^Department of Obstetrics and Gynecology, National Cheng Kung University Hospital, College of Medicine, National Cheng Kung University, Tainan, Taiwan

**Keywords:** stromal interaction molecule 1 (STIM1), store-operated Ca^2+^ entry (SOCE), direct stochastic optical reconstruction microscopy (dSTORM), microtubule network, somatic mutation

## Abstract

Store-operated Ca^2+^ entry (SOCE) is an essential pathway for Ca^2+^ signaling, and regulates various vital cellular functions. It is triggered by the endoplasmic reticulum Ca^2+^ sensor stromal interaction molecule 1 (STIM1). Illustration of STIM1 spatiotemporal structure at the nanometer scale during SOCE activation provides structural and functional insights into the fundamental Ca^2+^ homeostasis. In this study, we used direct stochastic optical reconstruction microscopy (dSTORM) to revisit the dynamic process of the interaction between STIM1, end-binding protein (EB), and microtubules to the ER-plasma membrane. Using dSTORM, we found that“powder-like”STIM1 aggregates into “trabecular-like” architectures toward the cell periphery during SOCE, and that an intact microtubule network and EB1 are essential for STIM1 trafficking. After thapsigargin treatment, STIM1 can interact with EB1 regardless of undergoing aggregation. We generated *STIM1* variants adapted from a real-world database and introduced them into SiHa cells to clarify the impact of *STIM1* mutations on cancer cell behavior. The p.D76G and p.D84Y variants locating on the Ca^2+^ binding domain of STIM1 result in inhibition of focal adhesion turnover, Ca^2+^ influx during SOCE and subsequent cell migration. Inversely, the p.R643C variant on the microtubule interacting domain of STIM1 leads to dissimilar consequence and aggravates cell migration. These findings imply that *STIM1* mutational patterns have an impact on cancer metastasis, and therefore could be either a prognostic marker or a novel therapeutic target to inhibit the malignant behavior of STIM1-mediated cancer cells. Altogether, we generated novel insight into the role of STIM1 during SOCE activation, and uncovered the impact of real-world *STIM1* variants on cancer cells.

## Introduction

Store-operated Ca^2+^ entry (SOCE), a major Ca^2+^ influx mechanism in most non-excitable cells, is an essential pathway for Ca^2+^ signaling (Cahalan, 2009). The pathway regulates vital cellular functions ranging from exocytosis, proliferation, and motility, to gene expression (Putney et al., 2017). The dynamic interactions between the pore subunits of the SOC channel, Orai1 and Orai3, and the endoplasmic reticulum (ER) Ca^2+^ sensors, stromal interaction molecule 1 (STIM1) and STIM2, constitute the key molecular mechanism of SOCE. STIM1 is an ER-resident transmembrane protein with several functional domains and protein-protein interaction motifs, which has a critical role in SOCE activation (Yeung et al., 2020). Several studies have reported the importance of the microtubule plus-end trafficking mechanism in the redistribution of STIM1 toward the ER-plasma membrane junctions, and the subsequent activation of Orai proteins (Chen et al., 2013a, 2019a). However, the ultrastructural organization of STIM1 during activation, aggregation, and translocation, as well as the interaction between STIM1, microtubules, and end-binding proteins (EBs) during SOCE remain unclear.

Some Ca^2+^-dependent molecules, such as calpain protease, myosin light-chain kinase and the focal adhesion proteins, including protein-rich tyrosine kinase (Pyk2), focal adhesion kinase (FAK), and talin, can be activated by STIM1-dependent Ca^2+^ influx (Chen et al., 2011, 2013a,2013b). STIM1-mediated SOCE impacts the behavior of tumor cells. By altering the focal adhesion turnover, actomyosin contractility, and invadopodia formation of cancer cells, the expression of STIM1 promotes tumorigenesis and tumor metastasis in different types of cancers (Chen et al., 2016, 2017, 2019b). However, the issue of whether the different genetic variants of *STIM1* lead to dissimilar consequences in the SOCE activation that modulates the behavior of cancer cells has not yet been studied.

In this study, we used direct stochastic optical reconstruction microscopy (dSTORM) to obtain detailed insight into STIM1 and the partners with which it interacts during SOCE. Compared to classical confocal microscopy, super-resolution dSTORM microscopy possesses high prospecting capacity by transcending the limitation of resolution (Hell, 2007; Huang et al., 2010; Xu et al., 2012), which allows us to revisit STIM1 protein in the scale of 20 nm. We also generated the real-world *STIM1* variants adapted from the Catalogue of Somatic Mutations in Cancer (COSMIC) database (Forbes et al., 2015; Tate et al., 2019) to study their impact on SOCE, and the subsequent alterations of cancer cell behaviors. We produced novel insights into STIM1 trafficking which is independent of aggregated state, and revealed the role of the microtubule network, end-binding protein EB1, and EB3 in SOCE. *STIM1* mutations result in alterations of Ca^2+^ influx, and subsequent cancer cell migration, which imply that STIM1 might be a potential prognostic marker and therapeutic target.

## Results

### Direct Stochastic Optical Reconstruction Microscopy Illustrated Super-Resolution Images of Stromal Interaction Molecule 1 During Activation

**FIGURE 1:**
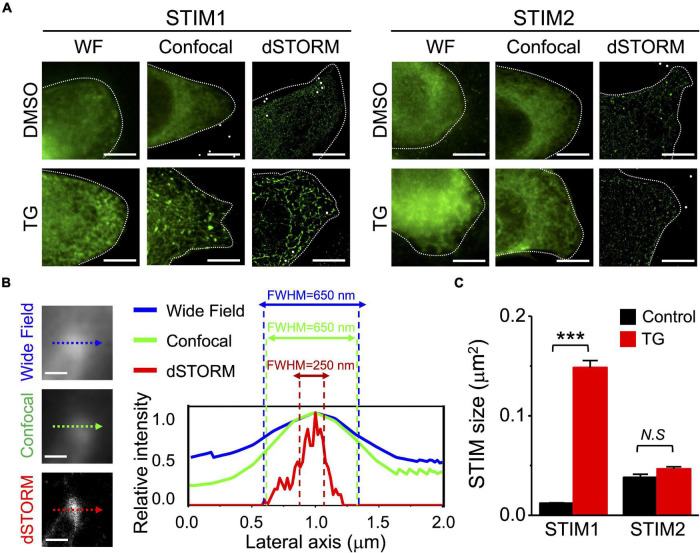
Visualization of the nanoscale architecture of STIM proteins in SiHa cervical cells overexpressing EGFP-STIM1 by confocal microscopy and dSTORM imaging. **(A)** STIM proteins (green) with or without thapsigargin (TG) stimulation (2 μM, 10 min) were imaged using wild-field (WF) epifluorescent, confocal microscopy, and super resolution direct stochastic optical reconstruction microscopy (dSTORM). Scale bar, 10 μm. Representative images are from at least six different cells of three different experiments. **(B)** SiHa cervical cells overexpressing EGFP-STIM1 were fixed for immunostaining for STIM1 after treatment with TG for 10 min. (Left), images of the same area in WF epifluorescent, confocal microscopy, and dSTORM. (Right), fluorescent intensity profiles along the dotted lines. We used full width at half maximum (FWHM) to measure the fluorescent intensities of STIM1 imaged by different methodologies. Scale bar, 0.2 μm. **(C)** Quantitative analyses of STIM molecular size using Integrated Morphometry Analysis (IMA) Measurement Parameters with MetaMorph Software (Supplementary Figure 3). Column, mean ± SEM from at least six cells of three independent experiments. ***P < 0.001, compared with one-way ANOVA with a Dunnett’s *post hoc* test. N.S., non-significant.

The ER Ca^2+^ sensors STIM1 and STIM2 are essential regulators of SOCE, and play important roles in the maintenance of intracellular Ca^2+^ homeostasis (Chen et al., 2019a). In the present study, we used SiHa cells overexpressing STIM1 or STIM2, treated with thapsigargin (TG) for ER Ca^2+^ depletion, to investigate the ultra-fine structure of STIM proteins during SOCE activation. The morphology of STIM1 overexpressed in SiHa cells after TG treatment is identical to the endogenous expression of STIM1 (Supplementary Figure 1), but the former is conductive for characterizing the architecture of STIM1 (Chen et al., 2011, 2013a). These structures were observed by fluorescence imaging with different techniques, including conventional wide-field (WF) epifluorescence microscopy, confocal microscopy, and super-resolution imaging by direct STORM (dSTORM) (Figure 1A). The estimated localization precision was demonstrated in Supplementary Figure 2 and Supplementary Movie 1. The full width at half maximum (FWHM) of STIM1 estimated by dSTORM imaging was ∼250 nm, much smaller than the ∼650 nm measured by WF and confocal microscopy (Figure 1B). Therefore, dSTORM imaging provided a more accurate characterization of STIM1 morphology compared to conventional imaging methodologies. According to confocal imaging, TG induced the aggregation of STIM1 into “puncta-like” structures (Figure 1A). In striking contrast, dSTORM showed that STIM1 exhibited “powder-like” structures scattered through the cytosol at resting status, and aggregated into “trabecular-like” architectures toward the cell periphery after 10 min of TG treatment (Figure 1A). The sizes of the activated STIM proteins were analyzed using Integrated Morphometry Analysis (IMA) Measurement Parameters and MetaMorph software (Molecular Devices, San Jose, CA, United States) (Figure 1B and Supplementary Figure 3). Compared to STIM1, STIM2 showed no obvious aggregation after TG treatment, either when assessed by confocal images or by dSTORM (Figure 1C).

**FIGURE 2:**
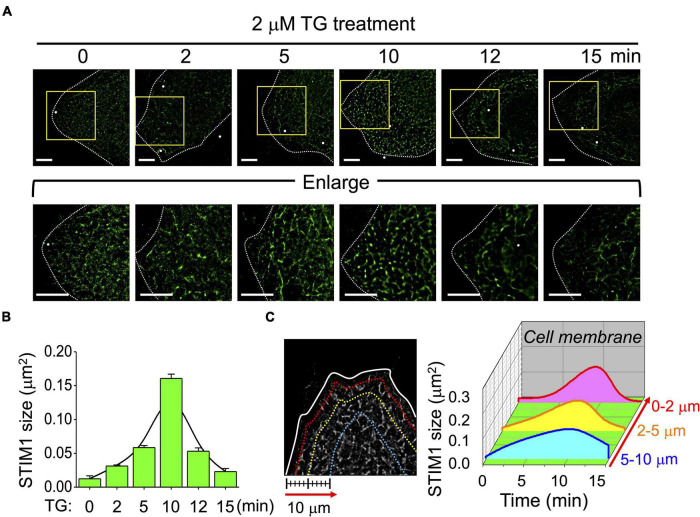
Spatiotemporal distribution of STIM1 during store-operated Ca^2+^ entry (SOCE) activation. **(A)** Representative dSTORM images of fixed cells at different time points of SOCE illustrating the intracellular distributions of STIM1 of SiHa cells upon TG treatment. Lower panels showing the enlargement of the areas indicated by rectangles in whole-cell images. Dashed line, cell periphery. White dots, TetraSpeck microspheres. Scale bar, 5 μm. **(B)** Quantitative analyses of STIM1 sizes at each indicated time point in TG-induced SOCE. Fitting curve, fitting a Gaussian curve to a bar graph. Each value represents mean ± SEM from at least six individual cells of three independent experiments. **(C)** (Left) The distribution of STIM1 proteins at 2, 5, and 10 μm from the cell periphery. (Right) Quantitative analyses of STIM1 proteins based on their intracellular areas and sizes. The *X*-axis indicated the time point after TG treatment. The *Y*-axis indicated the distribution of STIM1 proteins according to the distance from cell membrane. The *Z*-axis illustrated the average STIM1 sizes located in the indicated region at each time point. Data from more than six different cells of three independent experiments.

We also used dSTORM to observe the dynamic framework of STIM1 during SOCE. Figure 2A shows the time course of STIM1 activation induced by TG, indicating dynamic membrane trafficking. Figure 2B shows the quantitative analysis of STIM1 size during the activation and deactivation processes, which increased from 0.04 μm^2^ to a maximum of 0.15 μm^2^ after 10 min of TG treatment, and then decreased. We used dSTORM to map the spatiotemporal pattern of STIM1 spread in the cell following TG stimulation (Figure 2C and Supplementary Figure 3), and the most prominent alteration of STIM1 size was observed near the cell periphery, indicating the aggregation and trafficking of STIM1 to the plasma membrane during SOCE.

### Stromal Interaction Molecule 1 Membrane Trafficking Requires an Intact Microtubule Network

**FIGURE 3:**
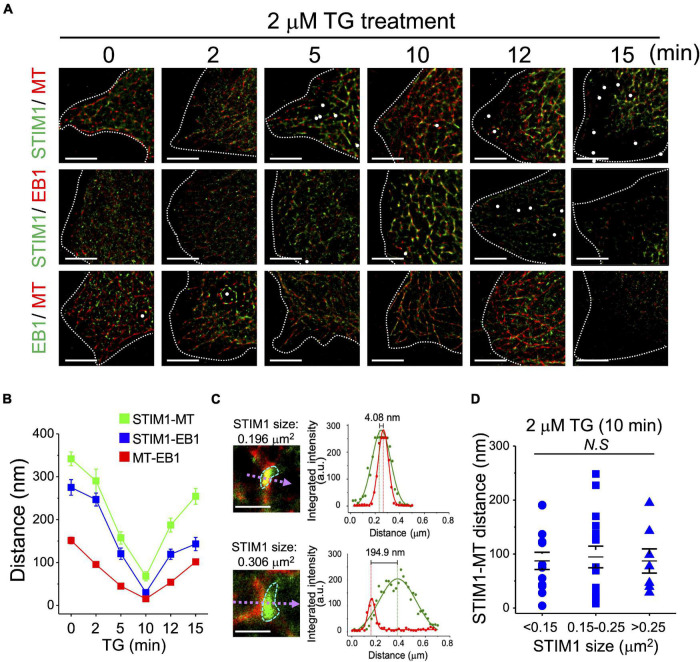
End-binding protein 1-dependent coupling of STIM1 and microtubules in SOCE activation. **(A)** Representative dSTORM images showing the intracellular distributions of STIM1, α-tubulin and EB1 in TG-induced SOCE of SiHa cells. Dashed line, cell periphery. White dots, TetraSpeck microspheres. Scale bar, 5 μm. **(B)** The distances between STIM1-α-tubulin, STIM1-EB, and α-tubulin-EB1 were analyzed using line fitting from the raw localization data. The full width at half-maximum (FWHM) was calculated from the resulting histogram, and calculated the molecular distance by minus the peak-to-peak distance (Supplementary Figure 4). Each value represents mean ± SEM from at least five cells of three independent experiments. **(C)** (Left) dSTORM images of STIM1 (green) and α-tubulin (red) after TG treatment quantified using MetaMorph. (Right) Dot, fluorescent intensity of STIM1 (green) and α-tubulin (red) along the dashed line in the ROI image. Line, Gaussian fit of fluorescent intensity. Dashed line, the highest fluorescent intensity, representing the molecular center. **(D)** Quantitative analyses of STIM1 size and STIM1-microtubule distance after 10 min of TG treatment. Each value represents mean ± SEM from at least six cells of three independent experiments. Compared by one-way ANOVA with a Dunnett’s *post hoc* test. N.S., non-significant.

**FIGURE 4:**
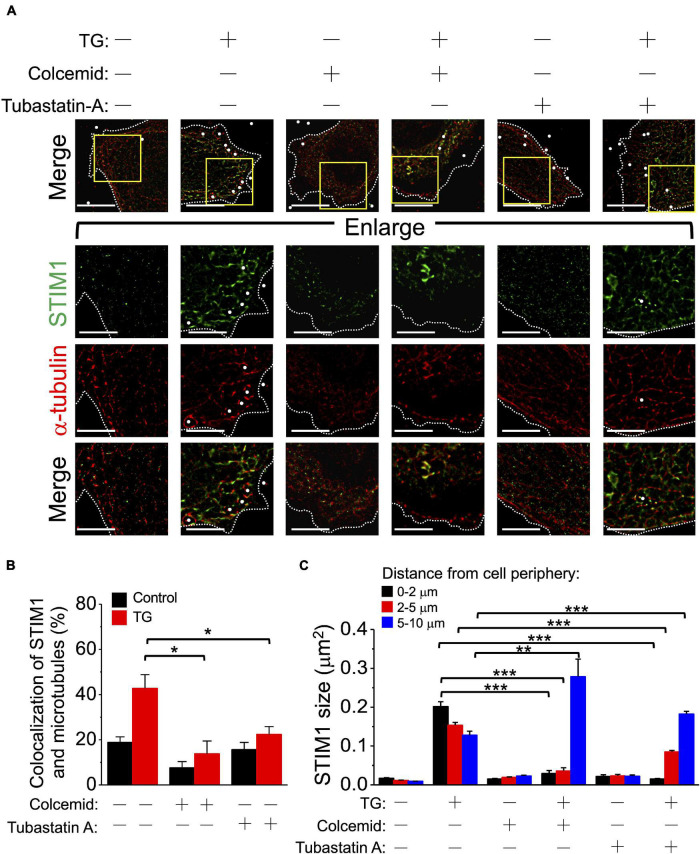
Tubastatin A and colcemid block STIM1 membrane trafficking but not STIM1 aggregation. **(A)** SiHa cells overexpressing EGFP-STIM1 were preincubated with 0.1% dimethyl sulfoxide for 5 h, 5 μM tubastatin A for 5 h, or 5 μg/mL colcemid for 30 min before TG (2 μM, 10 min) stimulation. Lower panels showing the enlargement of the areas indicated by rectangles in whole-cell images. Dashed line, cell periphery. White dots, TetraSpeck microspheres. Scale bar, 10 μm. **(B)** The co-localization ratio of STIM1 and microtubules at the juxta-plasma membrane area were quantified by pixel-by-pixel analyses. Column, mean ± SEM from at least five cells of three independent experiments. *P < 0.05, compared with control group by one-way ANOVA with a Dunnett’s *post hoc* test. N.S., non-significant. **(C)** The molecular sizes of STIM1 using dSTORM imaging were analyzed based on their distance from the cell periphery. Column, mean ± SEM from at least five cells of three independent experiments. **P < 0.01, ***P < 0.001, compared with control group by one-way ANOVA with a Dunnett’s *post hoc* test.

We have previously demonstrated the essential role of microtubules and end-binding protein 1 (EB1) for STIM1 trafficking (Chen et al., 2013a), and in this study we used dSTORM imaging to re-evaluate the functions of microtubules and EB1 during SOCE. Two-color mapping dSTORM imaging identified the morphological alterations and co-localization among STIM1, microtubules and EB1 in SOCE (Figure 3A). The molecular distance was calculated using FWHM according to the fluorescence intensity (Supplementary Figure 4). The distance between them was closest after TG treatment for 10 min (Figure 3B and Supplementary Table 1). The size of activated STIM1 could be variable while interacting with microtubules (Figures 3C,D), a finding which indicates that, once activated, STIM1 can bind to microtubules independent of aggregated state. This finding challenges the concept that only the STIM1 clusters interacts with microtubules (Liou et al., 2007; Soboloff et al., 2012; Chen et al., 2013a). We also used dSTORM imaging to clarify the framework of STIM2 during SOCE (Supplementary Figure 5). Although significant alterations were observed in the size and distance to microtubules of STIM1 after TG treatment, this phenomenon has not been observed in STIM2, an observation which implies that there was no significant aggregation or association between microtubules and STIM2 during SOCE. According to our previous study, the microtubule depolymerizer colcemid and the HDAC6 inhibitor tubastatin A both disrupted the microtubule network and subsequent SOCE (Chen et al., 2013a). In the present study, we used dSTORM imaging to assess this phenomenon. Except for colcemid and tubastatin A, we also applied another microtubule depolymerizer, nocodazole, to disrupt microtubules network, and which revealed that disruption of microtubule could inhibit the ER-plasma membrane trafficking of STIM1, but not aggregation, during SOCE (Figure 4 and Supplementary Figures 6, 7). The co-localization of STIM1 and microtubules was more disrupted by colcemid and tubastatin A than those between STIM1 and EB1 (Supplementary Figure 8). In summary, STIM1 therefore appears to interact with microtubules, regardless of undergoing aggregation, and the microtubule network is required for the ER-plasma membrane trafficking of STIM1.

### Deficiency of End-Binding Protein 1 and End-Binding Protein 3 Impairs Stromal Interaction Molecule 1 Trafficking and Decreases Store-Operated Ca^2+^ Entry

**FIGURE 5:**
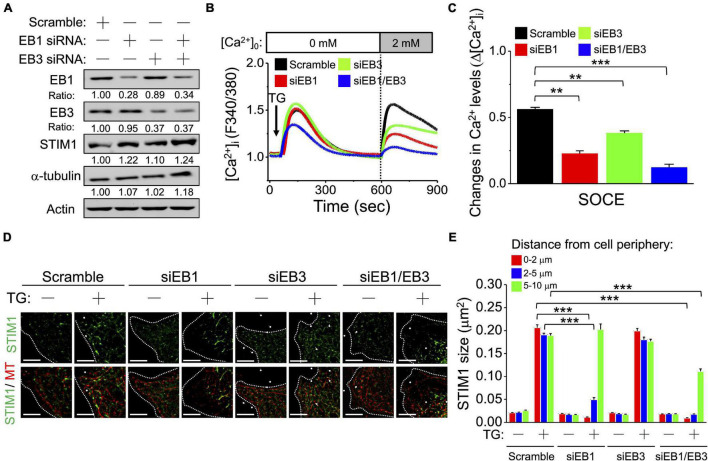
Microtubules plus end-binding proteins play important roles in regulating STIM1 trafficking. **(A)** EB1 and EB3 were silenced by siRNA. Representative immunoblots showing the expression levels of EB1, EB3, STIM1 and α-tubulin in SiHa cells. Expression of EB1, EB3, STIM1 and α-tubulin was normalized against β-actin. **(B)** The representative recordings from three different experiments to show [Ca^2+^]_i_ induced by 2 μM TG in extracellular Ca^2+^ ([Ca^2+^]_*o*_)-free media followed by replenishment of [Ca^2+^]_*o*_. The SOCE amplitude indicates the rise of [Ca^2+^]_i_ in replenishment of [Ca^2+^]_*o*_ from 0 to 2 mmol/L. Arrow, adding 2 μM TG. **(C)** Quantitative analyses of changes in Ca^2+^ levels (Δ[Ca^2+^]_i_). Column, mean ± SEM from three independent experiments, each independent experiment concluding at least 30 cells. **P < 0.01, ***P < 0.001, compared with control group by one-way ANOVA with a Dunnett’s *post hoc* test. **(D)** Representative images of cervical SiHa cancer cells overexpressing EGFP-STIM1 after EB1- or EB3-silencing for 72 h. Dashed line, cell periphery. White dots, TetraSpeck microspheres. Scale bar, 5 μm. **(E)** The molecular size of STIM1 by dSTORM imaging was analyzed based on their distance from cell periphery. Column, mean ± SEM from at least five different cells of three independent experiments. ***P < 0.001, compared with control group by one-way ANOVA with a Dunnett’s *post hoc* test.

As well as EB1, the microtubule plus-end binding protein 3 (EB3) has been reported to play an important role in the microtubule-dependent translocation and aggregation of STIM1 (Honnappa et al., 2009; Chen et al., 2019a). We used SiHa cells transfected with EB1 and EB3 small interfering RNA (siRNA) to study the impact of EB1 and EB3 on SOCE. Decreased expression level of EB1 and EB3 in the presence of EB1- and EB3-specific siRNA was confirmed using western blotting (Figure 5A and Supplementary Figure 9). After the downregulation of either EB1 or EB3, the change of cytosolic Ca^2+^ concentration (Δ[Ca^2+^]_i_) during SOCE was significantly decreased (Figures 5B,C). Two-color mapping dSTORM imaging demonstrated impairment of the microtubule network and accumulation of cytosol STIM1 (Figure 5D), which was a consequence of the defect in interactions between STIM1 and microtubules and in the ER-plasma membrane trafficking of STIM1 after the downregulation of EB1 (Figure 5E and Supplementary Figure 10). The interaction between STIM1 and the microtubule network were less strongly affected by siEB3 (Figures 5D,E). We examined the role of EB3 during SOCE after silencing EB1 (Supplementary Figure 11). Although the distance between STIM1 and EB3 was significantly decreased in the absence of EB1 (Supplementary Figure 11B), the impairment of STIM1 trafficking was still present. This finding implies that EB3 may partially compensate for the interactions between STIM1 and microtubules in the absence of EB1.

### Somatic Mutations of Stromal Interaction Molecule 1 Differentially Regulate Cancer Cell Migration by Changing Focal Adhesion Turnover

**FIGURE 6:**
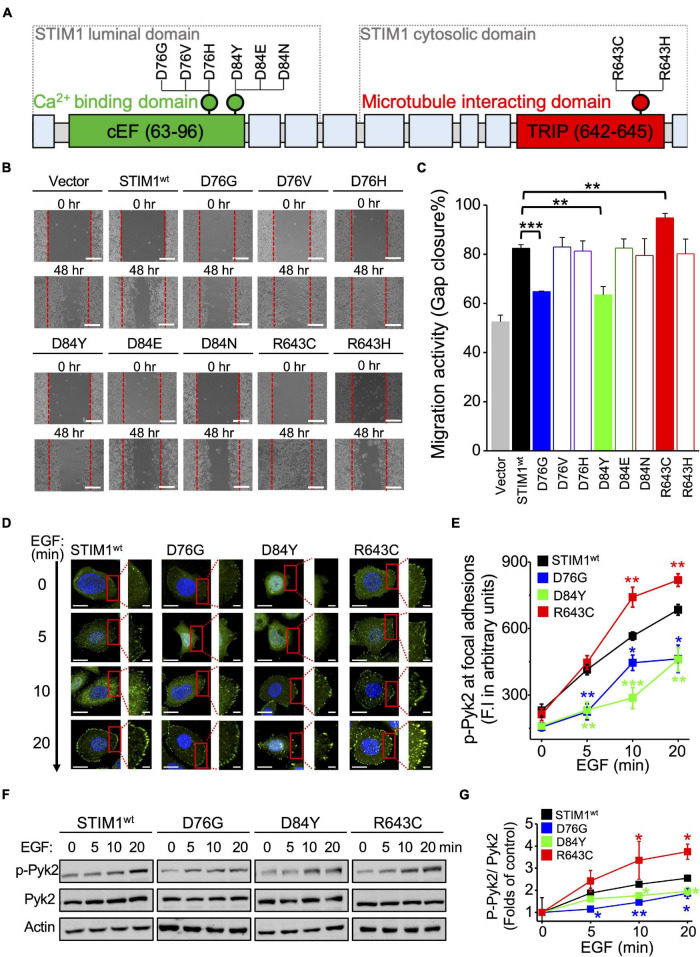
Somatic mutations in STIM1 differentially regulate cancer cell migration by focal adhesion turnover. **(A)** The molecular domains of STIM1 and the variants located on the cEF hand and S/TxIP motif. **(B)** Migratory activities of SiHa cells with different *STIM1* variants assessed by gap closure assay. Representative images at indicated time points from at least three independent experiments. The red dashed lines indicate where migration began. Scale bar, 200 μm. **(C)** Quantitative analyses of the migration activity of SiHa cells with different *STIM1* variants. Gap closure area quantified using the ImageJ software was taken as the index of cell migration activity. Column, mean ± SEM from at least three independent experiments. **P < 0.01, ***P < 0.001, compared with wild-type group by one-way ANOVA with a Dunnett’s *post hoc* test. **(D)** Representative images showing staining for p-Pyk2 (green) in SiHa cells with different *STIM1* variants. Scale bar, 10 μm. **(E)** Quantitative analyses of p-Pyk2 staining in SiHa cells with different *STIM1* variants. Column, mean ± SEM from at least five different cells of three independent experiments. *P < 0.05, **P < 0.01, ***P < 0.001, compared with wild-type group by one-way ANOVA with a Dunnett’s *post hoc* test. **(F)** The STIM1 mutations affect EGF-stimulated Pyk2 phosphorylation. Representative immunoblots from at least three independent experiments. **(G)** Densitometric quantification of Pyk2 phosphorylation (Tyr402) levels. Value, mean ± SEM from three independent experiments. *P < 0.05, **P < 0.01, compared with wild-type group by one-way ANOVA with a Dunnett’s *post hoc* test.

Stromal Interaction Molecule 1-dependent signaling plays an important role in cancer cell growth, migration, and angiogenesis (Chen et al., 2011). We aimed to uncover the impact of different *STIM1* variants on SOCE and cancer cell behavior. According to COSMIC, a real-world database, there are six somatic mutations in Ca^2+^ binding domains and two in microtubule interacting domains (Figure 6A and Supplementary Table 2). The six STIM1 variants were generated into SiHa cells using site-directed mutagenesis. The migration activity was decreased in the variants of the Ca^2+^ binding domain, p.D76G and p.D84Y, but was increased in the variant of the microtubule interacting domain p.R643C, compared to wild-type *STIM1* (Figures 6B,C). Pyk2 is uniquely located in focal adhesions and regulate cell migration. The Tyr402 autophosphorylation and focal adhesion targeting of Pyk2 are required for Pyk2-mediated cytoskeletal reorganization and the subsequent determination of focal adhesion turnover and cell migration (Mitra et al., 2006; Chen et al., 2011; Tsai et al., 2015). In consistent with the altered migration activity among different *STIM1* mutations, the level of pTyr402-Pyk2 level upon epidermal growth factor (EGF) stimulation in SiHa cells is inhibited in p.D76G and p.D84Y variants, but is stimulated in the p.R643C variant, compared to wild-type *STIM1* (Figures 6D–G). To sum up, these real-world *STIM1* somatic variants from the COSMIC database affect the migration activity of cancer cells by regulating the turnover of focal adhesion.

### Dissimilar Stromal Interaction Molecule 1 Dynamics in Different Variants Identified by Direct Stochastic Optical Reconstruction Microscopy

**FIGURE 7:**
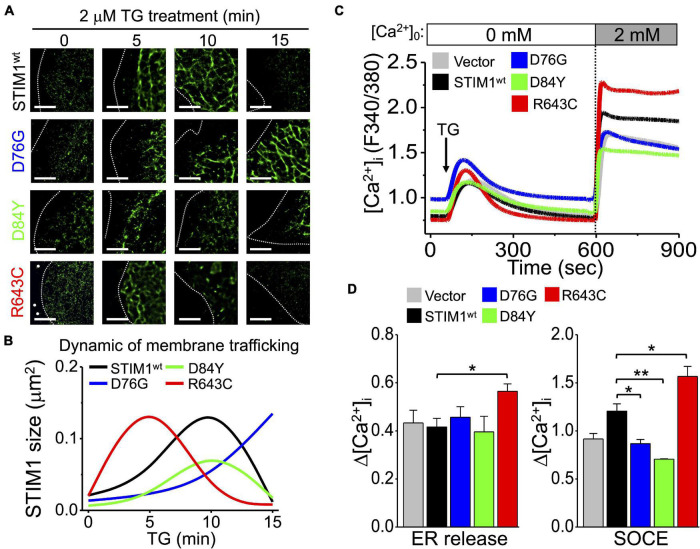
Mutations of STIM1 cEF hand and S/TxIP motifs affect the aggregation and trafficking of STIM1. **(A)** Representative dSTORM images showing the intracellular distributions of SiHa cells with different *STIM1* variants in TG-induced SOCE. Dashed line, cell periphery. White dots, TetraSpeck microspheres. Scale bar, 5 μm. **(B)** Quantitative analyses of SiHa cells with different *STIM1* variants based on the intracellular area and size of *STIM1* in TG-induced SOCE. Data from at least 30 ROIs from more than three different cells. **(C)** Mean traces of [Ca^2+^]_i_ measured from three independent experiments with different *STIM1* variants. Arrow, adding 2 μM TG. **(D)** (Left) Quantitative analyses of changes in ER Ca^2+^ levels (Δ[Ca^2+^]_i_) and (Right) quantitative analyses of changes in SOCE Ca^2+^ levels (Δ[Ca^2+^]_i_) in SiHa cells with different STIM1 variants. Column, mean ± SEM from three independent experiments, each independent experiment concluding at least 30 cells. *P < 0.05, **P < 0.01, compared with wild-type group by one-way ANOVA with a Dunnett’s *post hoc* test.

After establishing the impact of different *STIM1* variants on cancer cell migration, we further used dSTORM imaging to address STIM1 ER-plasma membrane trafficking in SiHa cells with p.D76G, p.D84Y, and p.R643C variants. There is distinct pattern of intracellular distribution and aggregation of STIM1 after TG treatment in SiHa cells harboring different variants (Figures 7A,B). The activation of STIM1 is inhibited in the p.D76G and p.D84Y variants but accelerated in the p.R643C variant. Similarly, the binding between STIM1 and microtubules is promoted in R643C variant (Supplementary Figure 12). The activity of SOCE is also consistent with the dynamic changes of STIM trafficking in wild-type and different *STIM1* variants (Figures 7C,D). In summary, different *STIM1* variants lead to dissimilar Ca^2+^ influxes during SOCE, which impact STIM1 dynamics and cancer cell migration (Figures 6, 7).

## Discussion

Direct stochastic optical reconstruction microscopy utilizes the photoswitching of a single fluorophore for imaging, and can produce an optical resolution of ∼20 nm. In this study, we used this technology to revisit the dynamic process of SOCE. The results provide new information about the ultrastructural organization of STIM1 during activation, aggregation, and translocation, as well as the interaction between STIM1, microtubules, and EBs during SOCE. First, instead of the “puncta-like” structure observed using confocal imaging, dSTORM revealed that activated STIM1 aggregates into a “trabecular-like” architecture while trafficking to the cell periphery. Interactions between microtubules and STIM1 can be initiated regardless of the aggregation or otherwise of STIM1 after TG treatment. These findings challenge the previous concept that biding to microtubules, and subsequent trafficking to the ER-plasma membrane junction, is followed by aggregation of STIM1. We also found that EB1 silencing did not impair aggregation, but trafficking of STIM1 to the ER-plasma membrane, and EB3 compensates for the crosstalk between STIM1 and microtubule after EB1-silencing.

The architecture of STIM1 has been illustrated by different imaging methodologies. Using the total internal reflection fluorescence (TIRF) microscopy (Perni et al., 2015) and Confocal microscopy (Li et al., 2017), TG induced STIM1 protein is shown to be aggregated into “plaques.” We have also previously demonstrated that TG induced STIM1 protein to be aggregated into “puncta” using confocal microscopy (Chen et al., 2013a) and TIRF microscopy in living cells (Chen et al., 2017). In the present study, we revisited the comprehensive molecular architecture of STIM1 using super-resolution microscopy. The various STIM1 architectures observed from different studies rise from the distinct imaging methodologies with varying degrees of resolution. Indeed, super-resolution microscopy could reach a spatial resolution of ∼20 nm or better in ideal situations, but the resolution of real-world images from immunostaining is usually less ideal. From the sizes of STIM1 puncta, it is still not clear whether the resolution is good enough to reliably distinguish STIM1 clusters from STIM1 single molecules. Moreover, because the information about the fluorescence intensity is lost in the reconstruction of dSTORM, the aggregation state of proteins cannot be inferred from puncta brightness. According to these images, an alternative interpretation would be that the extent of STIM1 aggregation is not strongly correlated with its ability to interact with microtubule, but aggregation may still be need.

Our previous study has indicated that deficiency of EB1 could inhibit STIM1 complexes-mediated ER movement in non-excitable cells, which proved that EB1 is necessary for SOCE activation and STIM1 membrane trafficking (Chen et al., 2013a), but so far there is a limited understanding of the role of EB3 during SOCE. Here, we demonstrated that microtubules play facilitative roles in the SOCE signaling pathway, and the deficiency of EB1 leads to impairment of STIM1 trafficking. On the contrary, EB3 knockdown results in the reduction of TG-mediate Ca^2+^ increase but not STIM1 trafficking, which implies that EB3 knockdown might interfere the coupling of STIM1 and Orai1 which impairs the SOCE. Smyth et al. (2007) documented a dependence of STIM1 localization on microtubules, wherein it was shown that microtubule depolymerization inhibit SOCE and nocodazole treatment does not prevent STIM1 reorganization into puncta. Chen and Honnappa indicated that STIM1 interact with the microtubule cytoskeleton through EB1 (Honnappa et al., 2009; Asanov et al., 2013; Chen et al., 2013a). However, some studies suggest that microtubules and EB1 are not necessary for the activation of SOCE. Grigoriev et al. (2008) showed that EB1 knockdown or the inhibition of microtubule dynamics by taxol had no significant influence on TG-induced SOCE in HeLa cells using fluorescence recovery after photobleaching. Tsai et al. (2014) demonstrated that a YFP-conjugated EB1-binding deficient mutant STIM-S1NN increased overall SOC influx to a similar degree as wild-type YFP-STIM1 in HUVEC. Some explanations might be responsible for these inconsistent findings. First, since the expression level of STIM1 and EB1 in different types of cells could be variable (Roos et al., 2005; DeHaven et al., 2007), the different cell lines applied in those studies might lead to distinct results. Moreover, the treatment duration of taxol in Grigoriev’s study last for only 2 h, and which is much shorter than the 24 h in other study (Dowdy et al., 2006; Zheng et al., 2016). Insufficient treatment might influence the effect of microtubule stabilization.

In the present study, we show that the distance of STIM1-EB1, STIM1-microtubule, and microtubule-EB1 all decreased during microtubule plus-end tracking of activated STIM1. The finding might be contributed from the conformational change for these three proteins to facilitate their interaction. Regarding the protein structure of EB1, the N-terminal calponin homology (CH) domains (aa 1–130) binds to the microtubule directly, while C-terminal EBC domains (aa 191–268) dimerize and serve as scaffolds for the other (+)TIPs (Akhmanova and Steinmetz, 2010). The conserved lysing/arginine (K/R) residues in the linker region of EB proteins are essential for their plus-end tracking property (Xia et al., 2014). Regarding the structure of STIM1, there is a binding domain for microtubule (S/TxIP motifs). Therefore, we suggest that these molecules might physically couple to form a complex to facilitate STIM1 trafficking. However, further studies are required to identify the critical role of the linker region of EB1 in tracking activated STIM1 clusters during SOCE activation.

We demonstrated that somatic mutations of STIM1 differentially regulate cancer cell migration by changing the turnover of focal adhesion. Several studies have shown that some disorders are associated with *STIM1* mutations which lead to immune dysregulation, such as non-syndromic tubular aggregate myopathy, York platelet syndrome and Stormorken syndromes (Lacruz and Feske, 2015; Cordero-Sanchez et al., 2019). These studies investigated the crucial role of SOCE in the immune system. They proposed that loss-of-function *STIM1* mutations resulted in impairment of Ca^2+^ influx resulting in defects of gene expression, cell growth and division, and immune dysfunction particularly NK-cell and T-cell inactivation, contributing to severe immunodeficiency (Feske et al., 2010). However, few studies have addressed the impact of *STIM1* mutations on cancer cell biology. We previously found that the expression level of STIM1 is significantly associated with the risk of cancer metastasis and survival, and blockade of SOCE activity inhibits tumor angiogenesis and growth in animal models (Chen et al., 2011). In this study, we transfected real-world *STIM1* variants located on Ca^2+^ binding or microtubule interacting domains to study their impacts on cancer cell migration. Different *STIM1* variants lead to dissimilar patterns of STIM1 aggregation and trafficking, and which were associated with alterations of Ca^2+^ influx, and subsequent cancer cell migration. For example, the mutation on microtubule interacting site (R643C) causes the increased ER calcium release, followed by upregulated calcium entry. However, the mutations on calcium binding sites (D76G, D84Y) do not affect ER calcium release but down-regulate the calcium entry. These results suggest the complexity on the mechanisms of STIM1-dependent SOCE activation, which are independent from the charge associate with the mutated residue. The STIM1 structure, especially S/TxIP motif, that regulating the binding of STIM1 to EB1 and microtubule remain unclear, and further structure analysis of STIM1 is needed. Importantly, the *STIM1* mutational pattern might have an impact on cancer metastasis and therefore might be a prognostic marker, and inhibitors of STIM1 are a potential treatment option for cancer patients.

In summary, we investigated the ultrastructure of STIM1 during SOCE and clarified its interactions with microtubule networks, EB1 and EB3 using dSTORM. We constructed a comprehensive molecular model of STIM1 activation, providing the structural and functional insights into the fundamental Ca^2+^ homeostasis, and uncovering the impact of *STIM1* variants on cancer cell behavior.

## Materials and Methods

### Cell Cultures and RNA Interference

SiHa human cervical cancer cells were maintained in Dulbecco’s modified Eagle medium High Glucose supplemented with 10% FBS and 2 mmol/L L-glutamine. The cells were maintained at 37°C in an environment containing 5% CO_2_. For the siRNA-mediated knockdown of EB1 or EB3, an siRNA pool of three different duplexes (Santa Cruz Biotechnology, Dallas, TX, United States) targeting EB1 or EB3 was used. Cells were transfected with 100 nM of either the targeting or the control siRNA (Santa Cruz Biotechnology) using Lipofectamine 2000 (Invitrogen, Carlsbad, CA, United States) for 48 or 72 h. The knockdown efficiency was determined by immunoblotting.

### Chemicals and Antibodies

Thapsigargin was obtained from Cayman Chemical (Ann Arbor, MI, United States). Colcemid was purchased from Merck (Darmstadt, Germany). Tubastatin A was obtained from BioVision (Milpitas, CA, United States). Nocodazole was purchased from Abcam (Cambridge, United Kingdom). Fura-2/AM was obtained from Invitrogen. EGF Recombinant Human Protein was purchased from Thermo Fisher (Thermo Fisher Scientific, Waltham, MA, United States). The antibodies used in this study are shown in Supplementary Table 3.

### Microscope Slide Preparation and Cell Seeding

Round glass coverslips 20 mm in diameter (No. 1.5, Paul Marienfeld GmbH, Thuringia, Germany) were cleaned using sonication for 15 min with 1 M aqueous potassium hydroxide, then dried. Before use, the slides were rinsed with 100% ethanol, dried, and coated with poly-D-lysine (SI-P0899, Sigma-Aldrich, St. Louis, MO, United States) for seeding cells. Fixed cells at different time points of SOCE were performed by dSTORM super-resolution microscopy.

### Direct Stochastic Optical Reconstruction Microscopy Super-Resolution Microscopy

Super-resolution microscopy in epifluorescence mode was performed using an IX81 inverted microscope (Olympus, Tokyo, Japan) equipped with the MetaMorph Microscopy Automation and Image Analysis Software (Molecular Devices, San Jose, CA, United States) with a super-resolution module using a 60× oil immersion objective (NA = 1.49). For dSTORM imaging, the exposure time was set as 50 ms, and the electron-multiplying gain as 100× (Leduc et al., 2018; Fan et al., 2020). Alexa Fluor 647 (Jackson ImmunoResearch Laboratories, West Grove, PA, United States) or CF 568 (Biotium, Fremont, CA, United States) fluorescence was bleached using the full laser power until individual fluorophores began to blink (Supplementary Figure 2 and Supplementary Movie 1). We imaged with exciting 561-nm laser following with 647-nm laser, which can avoid the bleaching of Alexa Flour 647 by 561-nm laser illumination. The 647-nm laser was used to excite fluorescence from Alexa Fluor 647 molecules. Before acquiring dSTORM images, we used relatively weak 647-nm light (∼0.05 W/cm^2^) to illuminate the sample and recorded the conventional fluorescence image before a substantial fraction of the dye molecules were switched off. We then increased the 647-nm light intensity (to ∼2 kW/cm^2^) to rapidly switch the dyes off for STORM imaging. The 561-nm laser followed the same steps. 15,000 frames were recorded to generate the final super-resolution image of the molecular ultrastructure. All imaging buffers were supplemented with the oxygen scavenging system, consisting of 10% (w/v) glucose (Sigma-Aldrich), 100 mM cysteamine (MEA) (M9768, Sigma-Aldrich), 313 mM glucose oxidase (G2133-250KU, Sigma-Aldrich), and 0.16 mM catalase from bovine liver (C40, Sigma-Aldrich). The oxygen scavenging system is critical for the reliable photoswitching of the fluorophores. For dSTORM imaging, 15,000 256 × 256 pixel frames were captured at with an electron multiplying charge coupled devices camera (Andor Technology, Belfast, United Kingdom). Image collection and fitting was performed using WaveTracer (MetaMorph, Molecular Devices, San Jose, CA, United States), which sets a photon-detection threshold to identify genuine blinking events, and uses a wavelet segmentation algorithm to fit a centroid to each blinking event within each 10 nm × 10 nm pixel. The point locations of individually blinking fluorophores in each of the 15,000 frames are collated to form a super-resolution image. TetraSpeck microspheres 0.1 μm on diameter (Thermo Fisher Scientific) were used to verify that there was no significant drift during image acquisition.

### Direct Stochastic Optical Reconstruction Microscopy Analysis

The super-resolution image was randomly selected from a single cell at a view area of 40 × 40 μm^2^ including cytosol and cell periphery in each experiment. To analyze STIM1 quantitatively, we used a DIC image to identify cell membrane and used MetaMorph software to define the distance from cell membrane in dSTORM imaging. With an appropriate and fixed threshold, STIM1 boundary and size were analyzed by IMA Measurement Parameters (Supplementary Figure 3). To measure the distance between two different molecules, we used line fitting from the raw localization data. We lined the region of interest (ROI) between two different molecules, each peak with a high enough signal-to-noise ratio was fitted to a Gaussian function by non-linear least-square fitting, positions as well as photon counts were extracted from the fitted peaks for rendering and quantification purposes (Olivier et al., 2013; Franke et al., 2017). The full width at half-maximum (FWHM) was calculated from the resulting histogram, and we calculated the molecular distance by minus the peak-to-peak distance (Supplementary Figure 4).

### Site-Directed Mutagenesis and Cell Transfection

The full-length cDNA of EGFP-STIM1 was kindly provided by Liangyi Chen of the Institute of Molecular Medicine, Peking University, China (Xu et al., 2006). pEX-SP-YFP-STIM1(23-685) (Addgene; #18857) was purchased from Addgene (Watertown, MA, United States). The mutant STIM1 was generated using site-directed mutagenesis kits (Agilent Technologies, Santa Clara, CA, United States). Primers for the site-directed mutagenesis of STIM1 variants were used to generate the cEF hand and S/TxIP mutant libraries (Supplementary Table 4). Reactions were amplified using the following protocol: 95°C denaturation for 2 min, followed by 20 cycles of denaturation at 95°C for 30 s, annealing at 60°C for 30 s, and extension at 68°C for 2.5 min, followed by a 10 min extension at 68°C. Three STIM1 sequencing primers were used for DNA sequencing to confirm the mutations: 5′-atggatgtatgcgtccgtcttgc-3′, 5′-atcacctcaaggacttcatgctggt-3′, and 5′-acagcattgcgggagc-3′. SiHa cervical cancer cells were transfected with plasmids using Polyplus-transfection reagents (Polyplus-Transfection SA, New York, NY, United States) and selected using G418 (Sigma-Aldrich). The stable pools of cells overexpressing STIM1 or STIM1 somatic variants were isolated using the MoFlo XDP Cell Sorter (Beckman Coulter, Brea, CA, United States).

### Databases for Analyzing Stromal Interaction Molecule 1 Somatic Variants

Stromal interaction molecule 1 variant analysis was carried out using the COSMIC database (Sondka et al., 2018). A lollipop diagram was created in trackViewer (Bioconductor software).

### Immunoblotting

For immunoblotting, cells were harvested with ice-cold modified radioimmunoprecipitation assay (RIPA) buffer containing a protease inhibitor cocktail (Roche Diagnostics, Basel, Switzerland), 100 mM potassium chloride, 80 mM sodium fluoride, 10 mM ethylene glycol tetraacetic acid, 50 mM h-glycerophosphate, 10 mM p-nitrophenyl phosphate, 1 mM vanadate, 0.5% sodium deoxycholate, and 1% NP40. Protein concentrations were determined with using a Bio-Rad protein assay (Bio-Rad Laboratories, Hercules, CA, United States). Equal amounts of protein lysates were separated by SDS-PAGE and then transferred to nitrocellulose blotting membranes (Pall Corporation, Port Washington, NY, United States). Immunoblots were blocked, incubated with the primary antibody, washed, and incubated with the corresponding horseradish peroxidase-conjugated secondary antibody (Jackson ImmunoResearch Laboratories, West Grove, PA, United States), and visualized using western blotting luminol reagent (Santa Cruz Biotechnology). Western blot bands were imaged and quantified using the iBright^*TM*^ FL1000 Imaging System (Thermo Fisher Scientific).

### Single Cell [Ca^2+^]_i_ Measurement

Cells attached on glass-bottom dishes were loaded with 2 μM Fura-2/AM in serum-free culture medium at 37°C for 30 min. Cells were then washed three times with phosphate buffered saline (PBS) before [Ca^2+^]_i_ measurement. ER Ca^2+^ release was induced with 2 μM TG in the absence of extracellular Ca^2+^, followed by the activation of SOCE with the addition of 2 mM Ca^2+^. The Fura-2 was excited alternatively between 340 nm (I340) and 380 nm (I380) using a Polychrome IV monochromator (Till Photonics GmbH, Gräfelfing, Germany) and images were detected using an inverted microscope (IX71, Olympus, Tokyo, Japan) equipped with a xenon illumination system and an IMAGO CCD camera (Till Photonics GmbH, Gräfelfing, Germany). The fluorescence intensity of excitation at 510 nm was monitored, to calculate [Ca^2+^]_i_ using HCImage Analysis (Hamamatsu Photonics, Shizuoka, Japan). The imaging parameters were acquired every 2 s per frame. We have used this protocol to measured SOCE in the series of studies on cervical cancer cells.

### Migration Assay

For the migration assays, the ibidi Culture-Insert 2 Well (ibidi GmbH, Planegg, Germany), which has two cell culture reservoirs separated by a 500 μm wall, was used. Cells were plated at 80,000 cells per well and allowed to attach overnight. On the following day, the culture inserts were removed, and light microscopy (IX71, Olympus, Tokyo, Japan) images were acquired. Cells were maintained under standard culture conditions while migrating toward the cell-free gap area, and images were acquired 48 h later. Images were analyzed using ImageJ software. The migration area was represented as the area remaining uncovered by cells.

### Immunofluorescence and Scanning Confocal Microscope

SiHa cervical cancer cells were fixed for 10 min at room temperature with 4% paraformaldehyde, or with methanol at −20°C for 10 min. Following fixation, the cells were washed two to three times in PBS, and then blocked with 3% BSA for 30 min at room temperature. Samples were incubated with primary antibodies in PBS at 4°C overnight and rinsed at least three times in PBS with Tween 20 (PBST), incubated with Alexa Fluor 647 secondary antibody (Jackson ImmunoResearch Laboratories, West Grove, PA, United States) for 1 h at room temperature, and rinsed at least three times in PBST. To detect the nucleus, cells were stained with Hoechst 33258 (Invitrogen) for 1 h at room temperature. The cells were then washed and mounted, and the fluorophores were excited by a laser at 405 nm or 640 nm, and detected using a scanning confocal microscope (FV-3000; Olympus, Tokyo, Japan). The fluorescence intensity of phosphor-Pyk2 (Tyr402) immunostaining was measured by the analysis program included in the FV-3000.

### Statistical Analysis and Reproducibility

Quantitative data are shown as mean ± SEM unless otherwise specified. The analyzed number (n) of samples is listed for each experiment.

## Data Availability Statement

The original contributions presented in the study are included in the article/Supplementary Material, further inquiries can be directed to the corresponding author/s.

## Author Contributions

All authors designed the research and analyzed the data. M-RS, Yi-TH, and Ya-TH performed the research and wrote the manuscript. Yi-TH contributed to analytic tools.

## Conflict of Interest

The authors declare that the research was conducted in the absence of any commercial or financial relationships that could be construed as a potential conflict of interest.

## Publisher’s Note

All claims expressed in this article are solely those of the authors and do not necessarily represent those of their affiliated organizations, or those of the publisher, the editors and the reviewers. Any product that may be evaluated in this article, or claim that may be made by its manufacturer, is not guaranteed or endorsed by the publisher.

## Supplementary Material

Supplementary Figure 1 The pathological significance of STIM1 overexpression can be proposed to benefit the locomotion of cancer cells. **(A)** SiHa mock cell line and **(B)** SiHa-EGFP-STIM1 cell line with or without TG stimulation (2 μM, 10 min) were imaged by wild-field (WF) epifluorescent and dSTORM. White dots, TetraSpeck microspheres. Scale bar, 10 μm.

Supplementary Figure 2 Evaluation of localization precision of the dSTORM system. **(A)** Measured photon distributions of Alexa Flour 647 and **(B)** CF 568 from fixed samples labeled with indicated markers. 15,000 frames were recorded to generate the final super-resolution image of the molecular ultrastructure.

Supplementary Figure 3 The steps of analyzing STIM1 sizes. **(A)** The steps to define the cell membrane by differential interference contrast (DIC). **(B)** Duplicate the analysis region and set the fixed and suitable threshold. STIM1 sizes were calculated by Integrated Morphometry Analysis (IMA) Measurement Parameters with MetaMorph software.

Supplementary Figure 4 Defined and analyzed the molecular distance of the dSTORM images. **(A)** Representative images and cross-sectional profiles for two nearby molecular. The white dotted one-way arrow indicates LineScan measured site. **(B)** The formula of Gaussian fit with width at half maximum (FWHM). **(C)** The molecular distances were calculated by Gaussian fit with FWHM, according to the fluorescence intensity. The two-way arrow indicates the peak-to-peak distance.

Supplementary Figure 5 Visualization of the nanoscale architecture of STIM proteins and microtubule network in SiHa cell using dSTORM imaging. **(A)** STIM proteins (green) and tubulin (red) with or without TG stimulation (2 μM, 10 min) were imaged by dSTORM. Lower panels were the enlarged view of corresponding dSTORM images. Dashed line, cell periphery. White dots, TetraSpeck microspheres. Scale bar, 10 μm. Representative images are from at least three different cells. **(B)** The distances between STIM and tubulin were analyzed by MetaMorph. Column, mean ± SEM from at least six different cells of three independent experiments. ****P* < 0.001, compared with control group by one-way ANOVA with a Dunnett’s *post hoc* test. N.S., non-significant.

Supplementary Figure 6 Microtubule depolymerizer inhibitor, Nocodazole, block STIM1 membrane trafficking but not STIM1 aggregation. **(A)** SiHa cells overexpressing EGFP-STIM1 were preincubated with 0.1% dimethyl sulfoxide for 1 h or 10 μg/mL nocodazole for 1 h before TG (2 μM, 10 min) stimulation. Dashed line, cell periphery. White dots, TetraSpeck microspheres. Scale bar, 10 μm. **(B)** The molecular sizes of STIM1 using dSTORM imaging were analyzed based on their distance from the cell periphery. Column, mean ± SEM from at least six different cells of three independent experiments. **P* < 0.05, compared with control group by one-way ANOVA with a Dunnett’s *post hoc* test. **(C)** The molecular size of STIM1 by dSTORM imaging was analyzed based on their distance from cell periphery. Column, mean ± SEM from at least five different cells of three independent experiments. ****P* < 0.001, compared with control group by one-way ANOVA with a Dunnett’s *post hoc* test.

Supplementary Figure 7 HDAC6 inhibitor, Tubastatin A, block STIM1 membrane trafficking but not STIM1 aggregation. SiHa cervical cancer cells overexpressing EGFP-STIM1 were preincubated with 0.1% dimethyl sulfoxide or 5 μM tubastatin A for 5 h before TG (2 μM, 10 min) stimulation. Lower panels showing the enlargement of the areas indicated by rectangles in whole-cell images. Dashed line, cell periphery. White dots, TetraSpeck microspheres. Scale bar, 10 μm.

Supplementary Figure 8 Tubastatin A and colcemid inhibit EB1-dependent coupling of STIM1 with microtubule in SOCE activation. **(A)** SiHa cervical cancer cells overexpressing EGFP-STIM1 were preincubated with 0.1% dimethyl sulfoxide, 5 μM tubastatin A for 5 h, or 5 μg/mL colcemid for 30 min before TG (2 μM, 10 min) stimulation. Lower panels showing the enlargement of the areas indicated by rectangles in whole-cell images. Dashed line, cell periphery. White dots, TetraSpeck microspheres. Scale bar, 10 μm. **(B)** The co-localization ratio of STIM1 and EB1 at the juxta-plasma membrane area was quantified by pixel-by-pixel analyses. Column, mean ± SEM from at least six different cells of three independent experiments. ^∗^*P* < 0.05, ^∗∗^*P* < 0.01, compared with control group by one-way ANOVA with a Dunnett’s *post hoc* test.

Supplementary Figure 9 The siRNA knockdown efficiency was evaluated by testing the protein level of EB1 and EB3. **(A)** Western blot analysis of EB1 and **(B)** EB3 protein level in SiHa cells 72 h after silenced by siRNA. Column, mean ± SEM from three independent experiments. ***P* < 0.01, ****P* < 0.001, compared with control group by one-way ANOVA with a Dunnett’s *post hoc* test.

Supplementary Figure 10 The defect in interactions between STIM1 and microtubules after the downregulation of EB1. **(A)** The co-localization ratio of STIM1 and EB1 at the juxta-plasma membrane area was quantified by pixel-by-pixel analyses. Column, mean ± SEM from at least six different cells of three independent experiments. **P* < 0.05, ***P* < 0.01, ****P* < 0.001, compared with control group by one-way ANOVA with a Dunnett’s *post hoc* test. **(B)** Quantitative analyses of the molecular distances between STIM1 and microtubules with or without EB1 or EB3 silencing. Column, mean ± SEM from at least six different cells of three independent experiments. ****P* < 0.001, compared with control group by one-way ANOVA with a Dunnett’s *post hoc* test.

Supplementary Figure 11 Microtubule plus end-binding proteins play a compensatory role in regulating STIM1 trafficking. **(A)** Representative images showing that SiHa cells overexpressing EGFP-STIM1 were silenced for EB1 or not for 72 h. Lower panels showing the enlargement of the areas indicated by rectangles in whole-cell images. Dashed line, cell periphery. White dots, TetraSpeck microspheres. Scale bar, 10 μm. **(B)** Quantitative analyses of the molecular distances between STIM1 and EB3 with or without siEB1. Column, mean ± SEM from at least five different cells of three independent experiments. ***P* < 0.01, ****P* < 0.001, compared with control group by one-way ANOVA with a Dunnett’s *post hoc* test.

Supplementary Figure 12 Two mutations of STIM1 on S/TxIP motifs displayed distinguished cellular function. **(A)** Representative dSTORM images showing the intracellular distributions of SiHa cells with different *STIM1* variants in TG-induced SOCE. Dashed line, cell periphery. White dots, TetraSpeck microspheres. Scale bar, 5 μm. **(B)** Quantitative analyses of the distances between SiHa cells with different *STIM1* variants and microtubules. Each value represents the mean ± SEM from at least 30 ROIs of three independent experiments. **P* < 0.05, ***P* < 0.01, ****P* < 0.001, compared with wild-type group by one-way ANOVA with a Dunnett’s *post hoc* test.

## Abbreviations

STIM: stromal interaction molecule
SOCE: store-operated Ca^2+^ entry
ER: endoplasmic reticulum
dSTORM: direct stochastic optical reconstruction microscopy
EB: end-binding protein
Pyk2: protein-rich tyrosine kinase 2
FAK: focal adhesion kinase
COSMIC: Catalogue of Somatic Mutations in Cancer
WF: wide-field
FWHM: full width at half maximum
TG: thapsigargin
siRNA: small interfering RNA
[Ca^2+^]_i_: intracellular Ca^2+^
EGF: epidermal growth factor
RIPA: radioimmunoprecipitation assay
PBS: phosphate buffered saline.
