# Supplementary figures and images for: Super-Resolution Microscopy Reveals That Stromal Interaction Molecule 1 Trafficking Depends on Microtubule Dynamics
Source: Front Physiol. 2021 Nov 5;12:762387. doi: 10.3389/fphys.2021.762387 (PMC8602801; doi:10.3389/fphys.2021.762387)

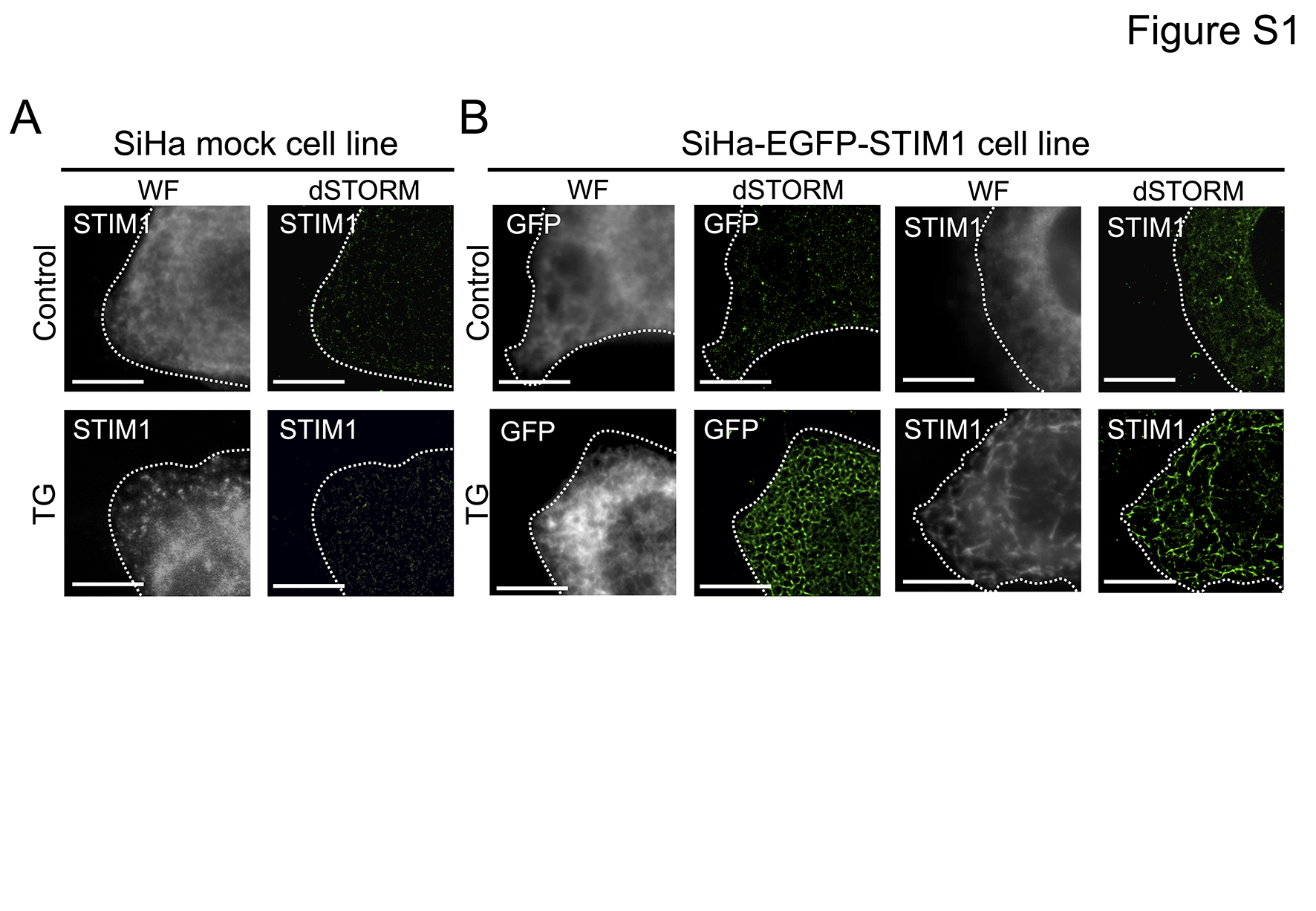

Supplement: Supplementary Figure 1 — The pathological significance of STIM1 overexpression can be proposed to benefit the locomotion of cancer cells. (A) SiHa mock cell line and (B) SiHa-EGFP-STIM1 cell line with or without TG stimulation (2 μM, 10 min) were imaged by wild-field (WF) epifluorescent and dSTORM. White dots, TetraSpeck microspheres. Scale bar, 10 μm. [file Image_1.TIFF]

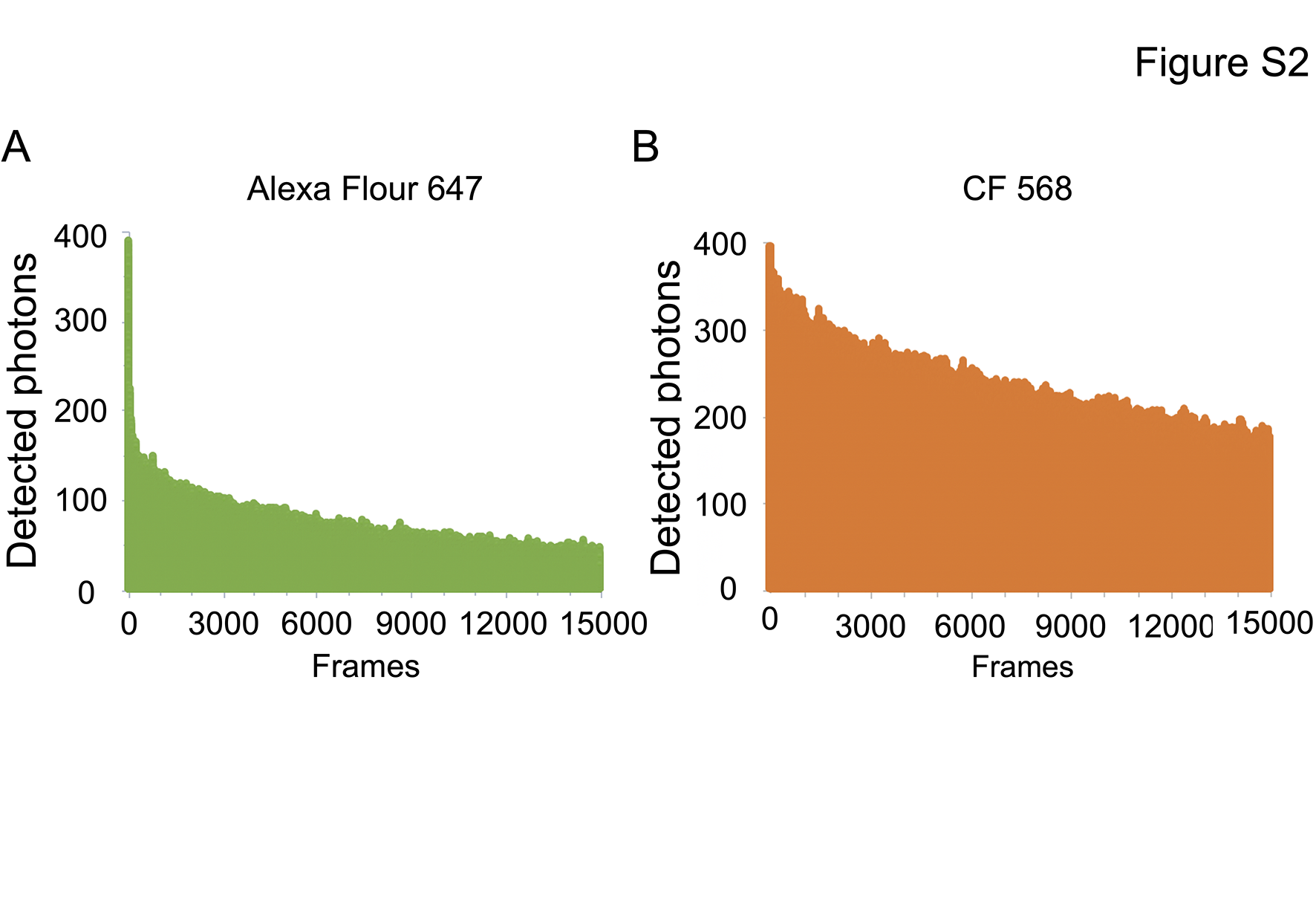

Supplement: Supplementary Figure 2 — Evaluation of localization precision of the dSTORM system. (A) Measured photon distributions of Alexa Flour 647 and (B) CF 568 from fixed samples labeled with indicated markers. 15,000 frames were recorded to generate the final super-resolution image of the molecular ultrastructure. [file Image_2.TIFF]

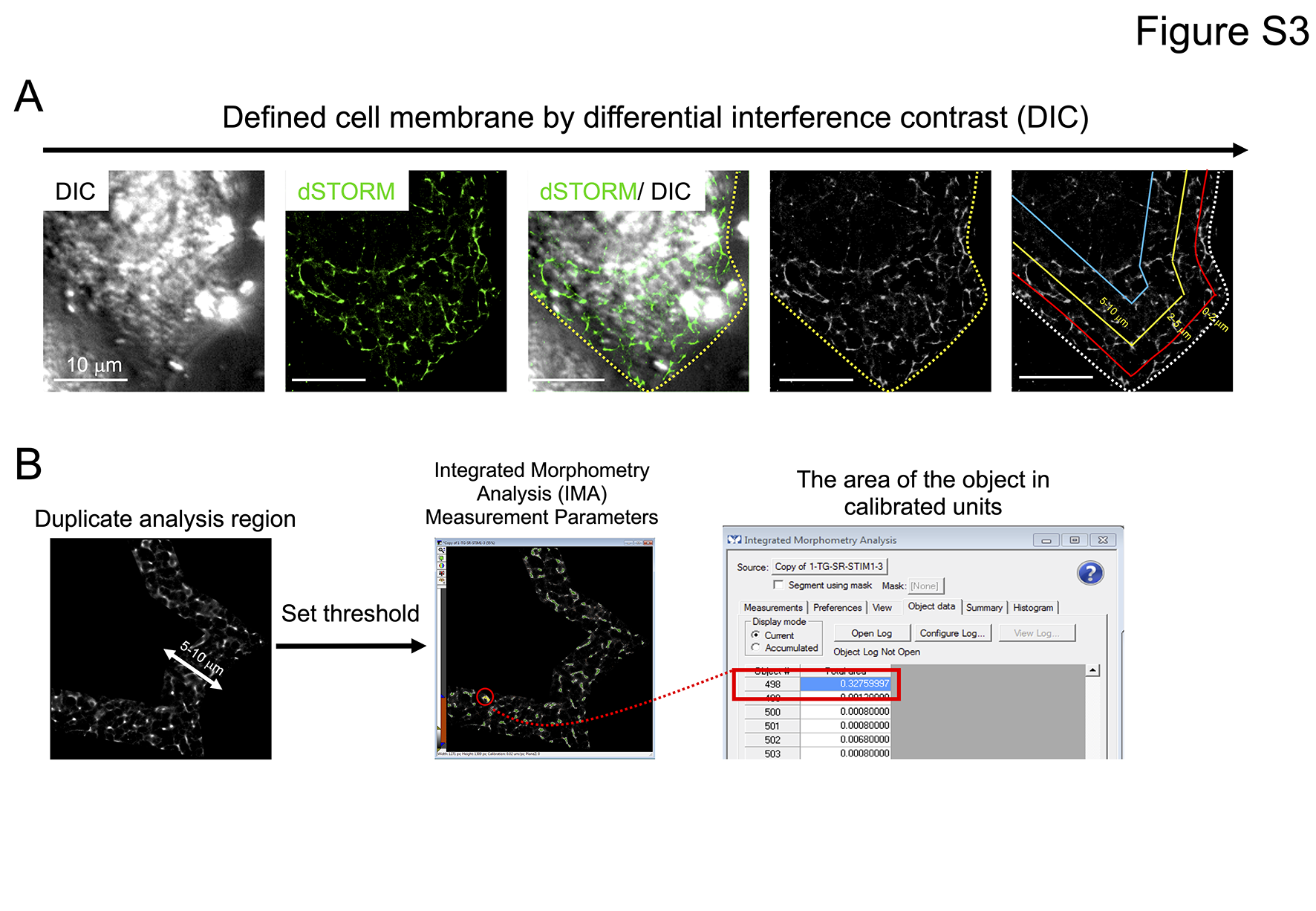

Supplement: Supplementary Figure 3 — The steps of analyzing STIM1 sizes. (A) The steps to define the cell membrane by differential interference contrast (DIC). (B) Duplicate the analysis region and set the fixed and suitable threshold. STIM1 sizes were calculated by Integrated Morphometry Analysis (IMA) Measurement Parameters with MetaMorph software. [file Image_3.TIFF]

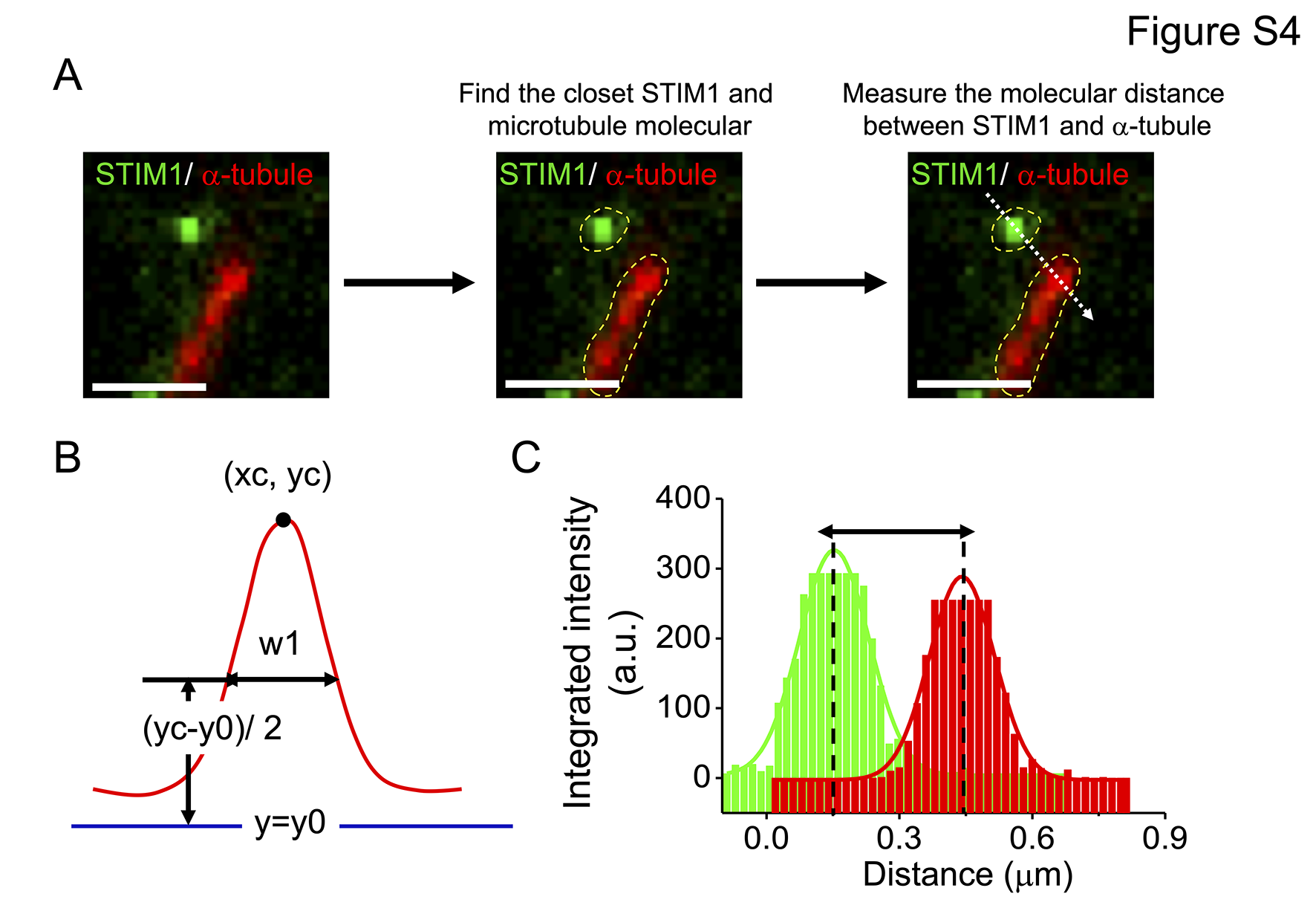

Supplement: Supplementary Figure 4 — Defined and analyzed the molecular distance of the dSTORM images. (A) Representative images and cross-sectional profiles for two nearby molecular. The white dotted one-way arrow indicates LineScan measured site. (B) The formula of Gaussian fit with width at half maximum (FWHM). (C) The molecular distances were calculated by Gaussian fit with FWHM, according to the fluorescence intensity. The two-way arrow indicates the peak-to-peak distance. [file Image_4.TIFF]

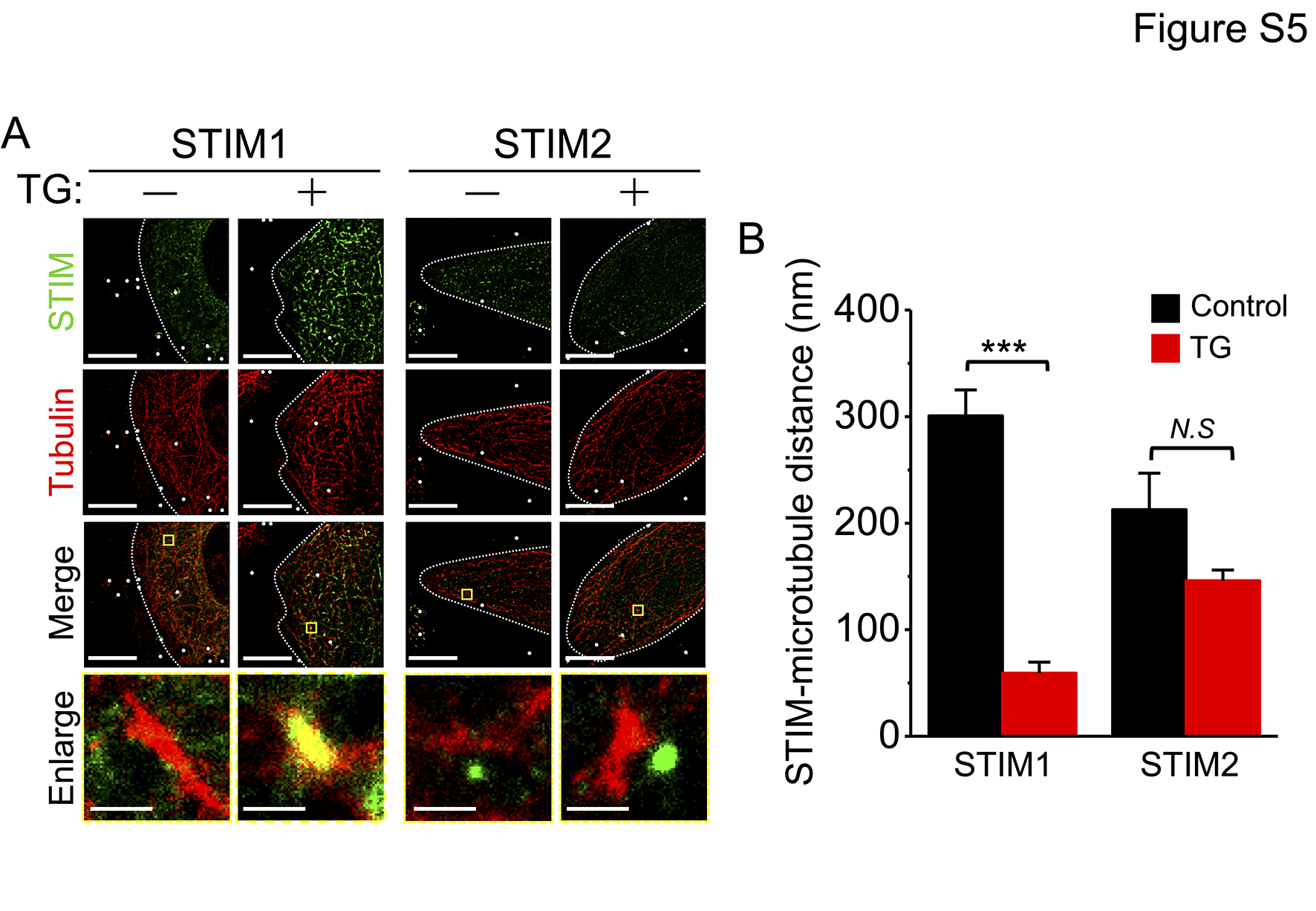

Supplement: Supplementary Figure 5 — Visualization of the nanoscale architecture of STIM proteins and microtubule network in SiHa cell using dSTORM imaging. (A) STIM proteins (green) and tubulin (red) with or without TG stimulation (2 μM, 10 min) were imaged by dSTORM. Lower panels were the enlarged view of corresponding dSTORM images. Dashed line, cell periphery. White dots, TetraSpeck microspheres. Scale bar, 10 μm. Representative images are from at least three different cells. (B) The distances between STIM and tubulin were analyzed by MetaMorph. Column, mean ± SEM from at least six different cells of three independent experiments. ***P < 0.001, compared with control group by one-way ANOVA with a Dunnett’s post hoc test. N.S., non-significant. [file Image_5.TIFF]

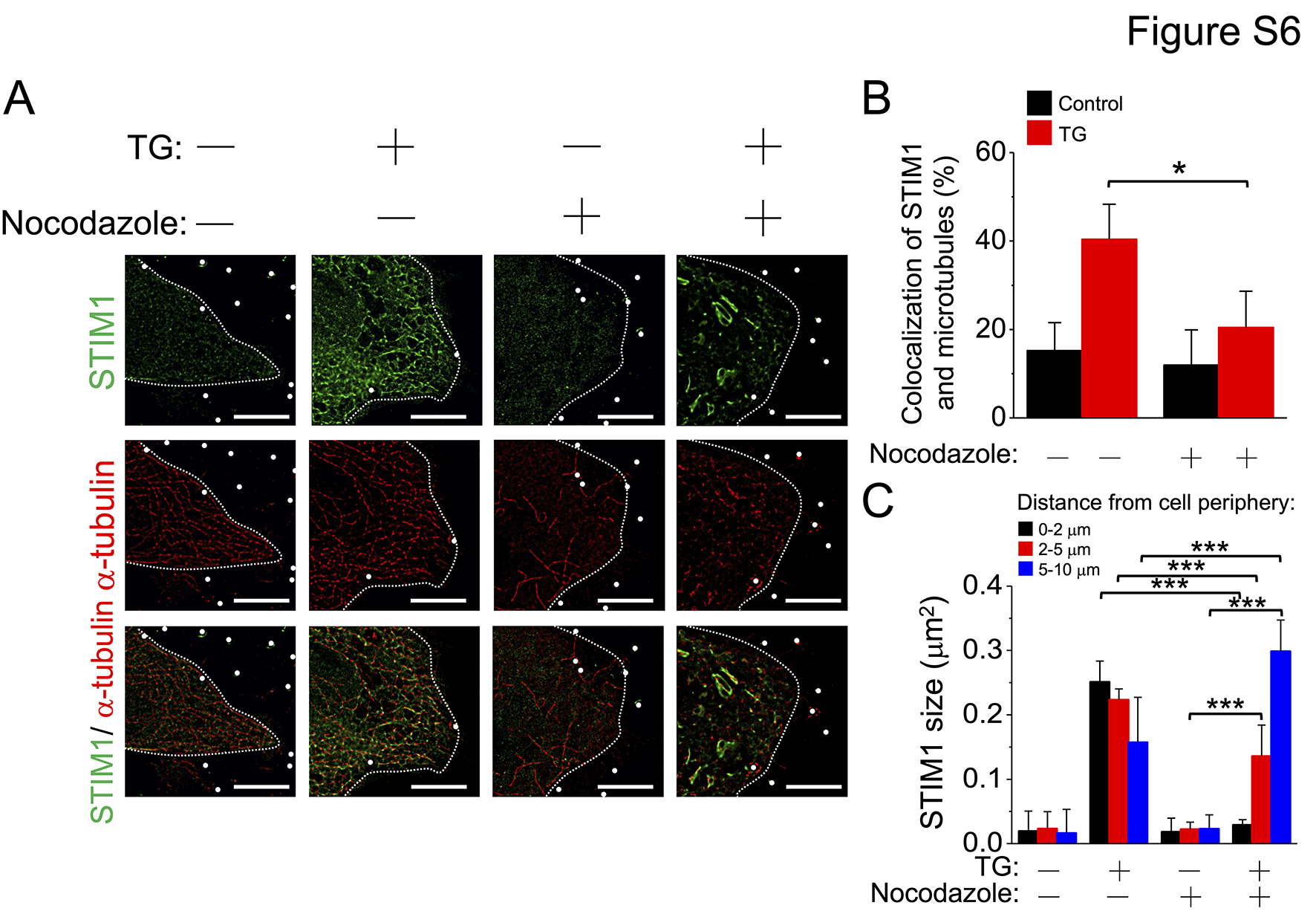

Supplement: Supplementary Figure 6 — Microtubule depolymerizer inhibitor, Nocodazole, block STIM1 membrane trafficking but not STIM1 aggregation. (A) SiHa cells overexpressing EGFP-STIM1 were preincubated with 0.1% dimethyl sulfoxide for 1 h or 10 μg/mL nocodazole for 1 h before TG (2 μM, 10 min) stimulation. Dashed line, cell periphery. White dots, TetraSpeck microspheres. Scale bar, 10 μm. (B) The molecular sizes of STIM1 using dSTORM imaging were analyzed based on their distance from the cell periphery. Column, mean ± SEM from at least six different cells of three independent experiments. *P < 0.05, compared with control group by one-way ANOVA with a Dunnett’s post hoc test. (C) The molecular size of STIM1 by dSTORM imaging was analyzed based on their distance from cell periphery. Column, mean ± SEM from at least five different cells of three independent experiments. ***P < 0.001, compared with control group by one-way ANOVA with a Dunnett’s post hoc test. [file Image_6.TIFF]

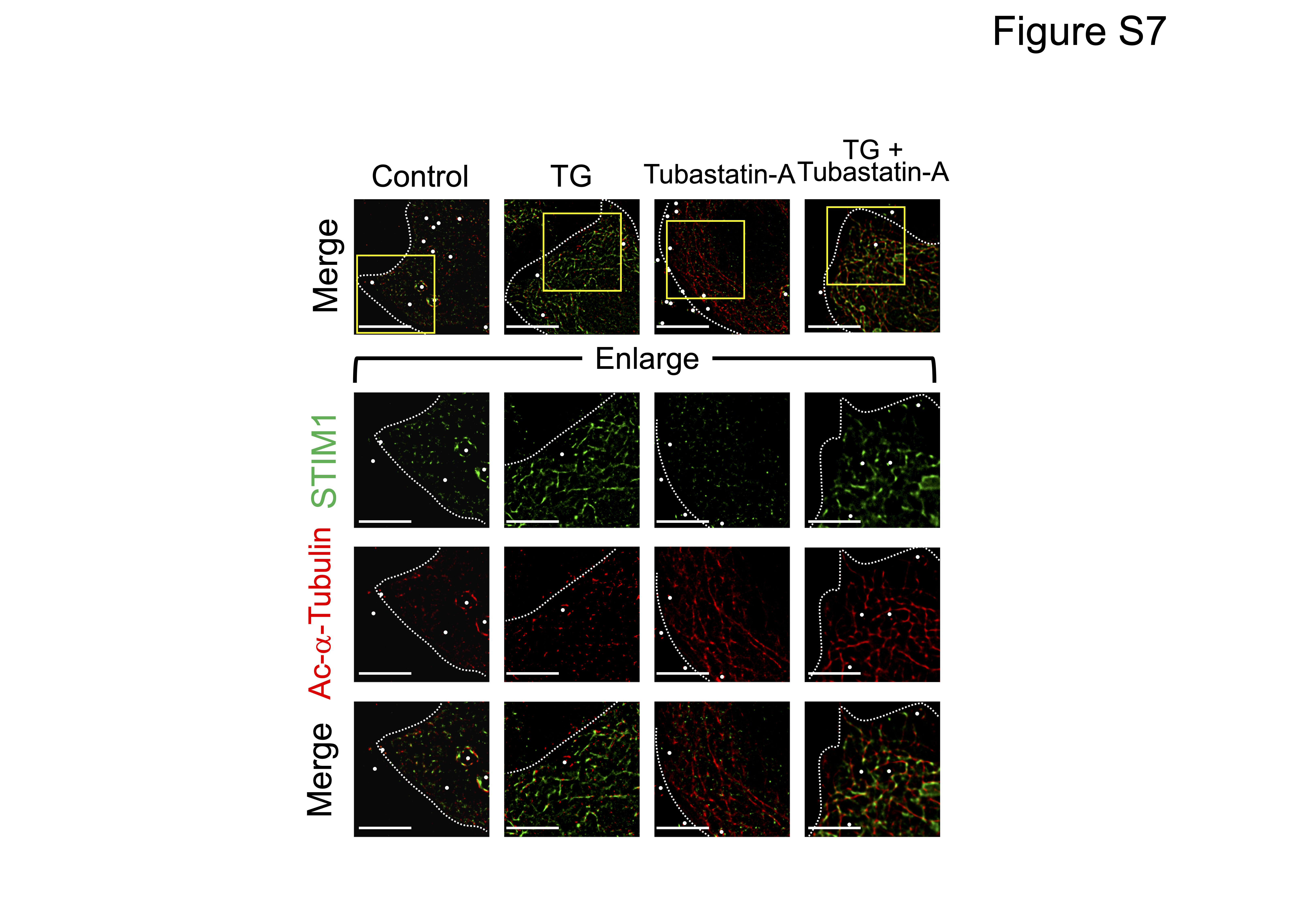

Supplement: Supplementary Figure 7 — HDAC6 inhibitor, Tubastatin A, block STIM1 membrane trafficking but not STIM1 aggregation. SiHa cervical cancer cells overexpressing EGFP-STIM1 were preincubated with 0.1% dimethyl sulfoxide or 5 μM tubastatin A for 5 h before TG (2 μM, 10 min) stimulation. Lower panels showing the enlargement of the areas indicated by rectangles in whole-cell images. Dashed line, cell periphery. White dots, TetraSpeck microspheres. Scale bar, 10 μm. [file Image_7.tiff]

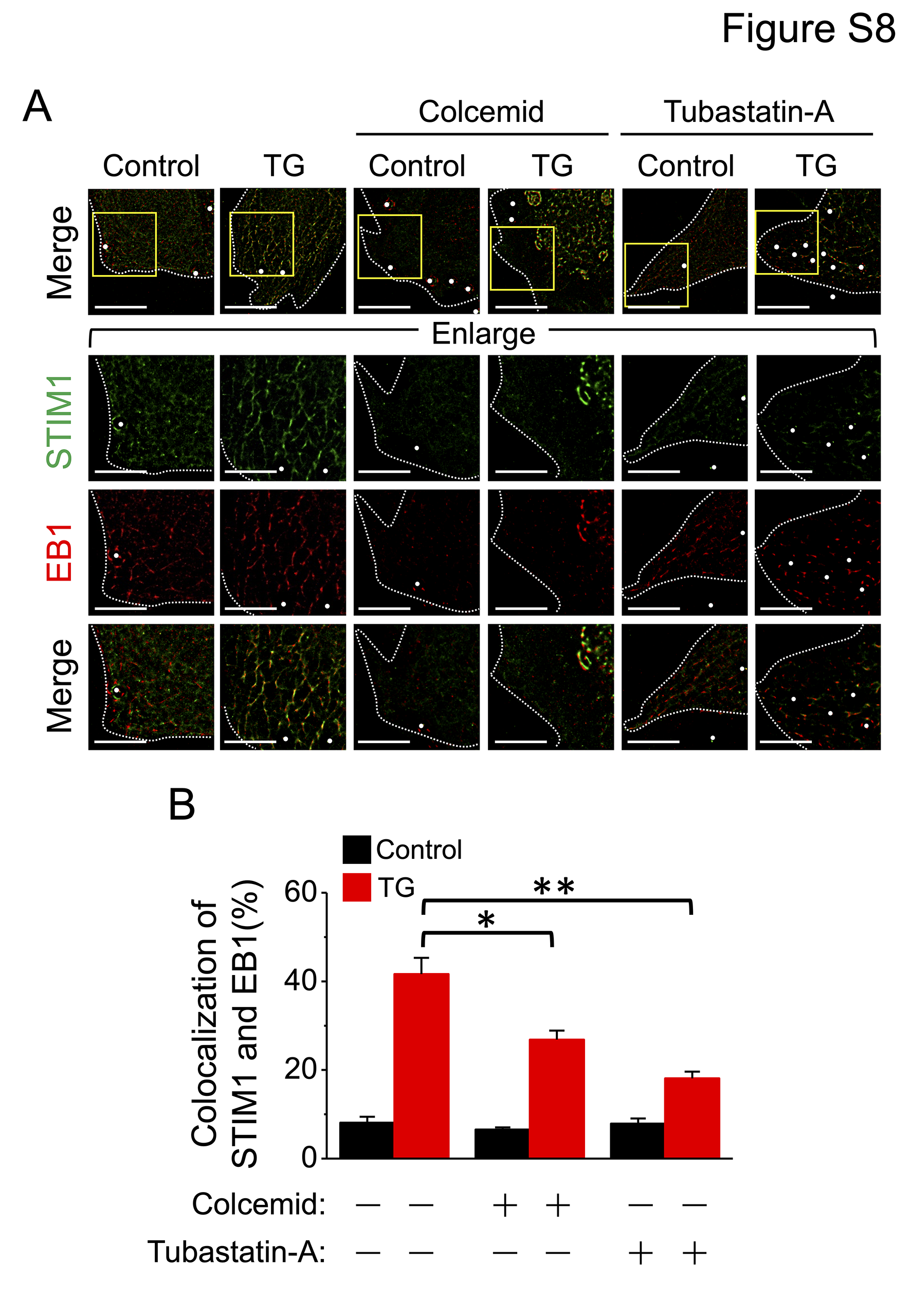

Supplement: Supplementary Figure 8 — Tubastatin A and colcemid inhibit EB1-dependent coupling of STIM1 with microtubule in SOCE activation. (A) SiHa cervical cancer cells overexpressing EGFP-STIM1 were preincubated with 0.1% dimethyl sulfoxide, 5 μM tubastatin A for 5 h, or 5 μg/mL colcemid for 30 min before TG (2 μM, 10 min) stimulation. Lower panels showing the enlargement of the areas indicated by rectangles in whole-cell images. Dashed line, cell periphery. White dots, TetraSpeck microspheres. Scale bar, 10 μm. (B) The co-localization ratio of STIM1 and EB1 at the juxta-plasma membrane area was quantified by pixel-by-pixel analyses. Column, mean ± SEM from at least six different cells of three independent experiments. ∗P < 0.05, ∗∗P < 0.01, compared with control group by one-way ANOVA with a Dunnett’s post hoc test. [file Image_8.TIFF]

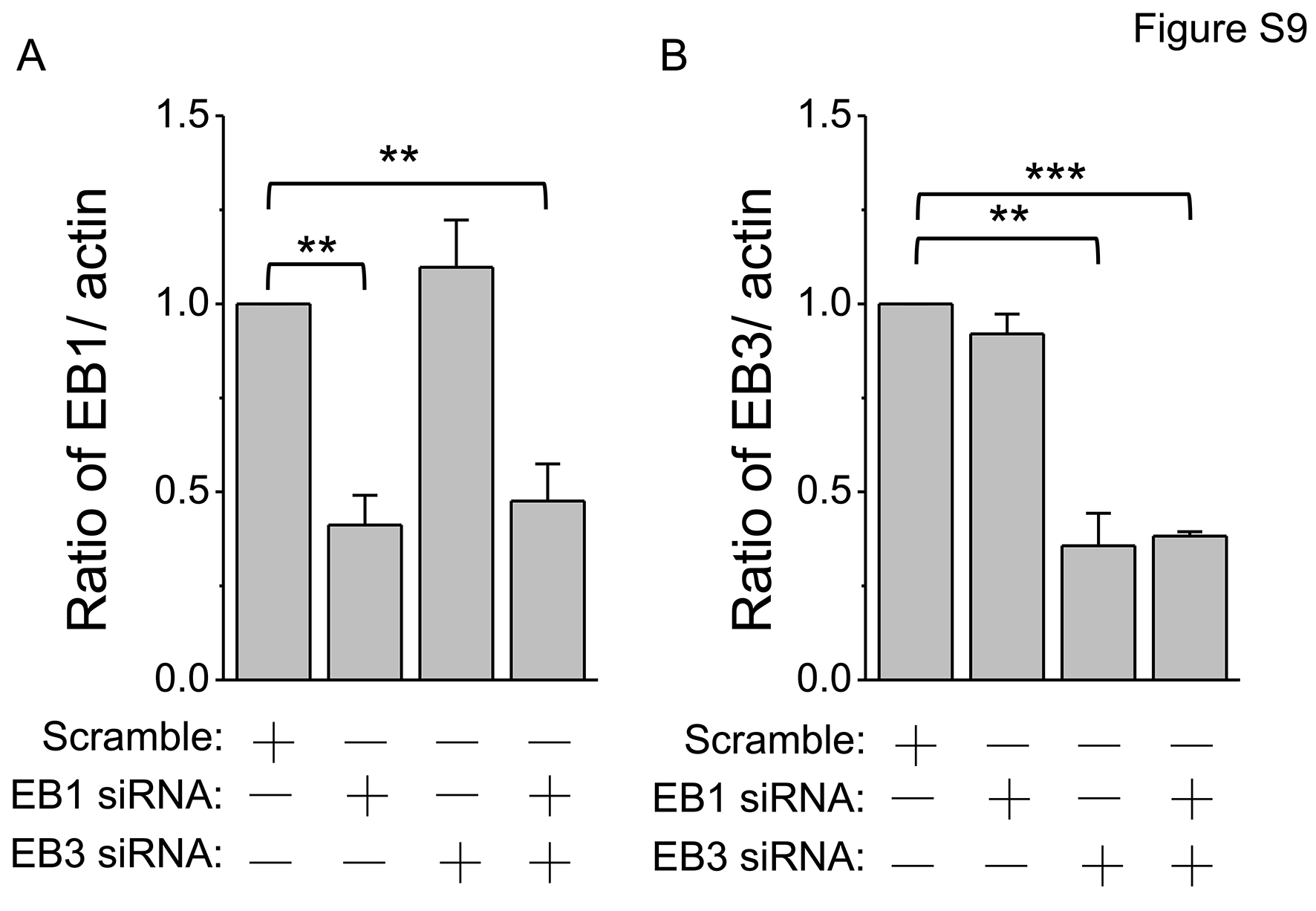

Supplement: Supplementary Figure 9 — The siRNA knockdown efficiency was evaluated by testing the protein level of EB1 and EB3. (A) Western blot analysis of EB1 and (B) EB3 protein level in SiHa cells 72 h after silenced by siRNA. Column, mean ± SEM from three independent experiments. **P < 0.01, ***P < 0.001, compared with control group by one-way ANOVA with a Dunnett’s post hoc test. [file Image_9.TIFF]

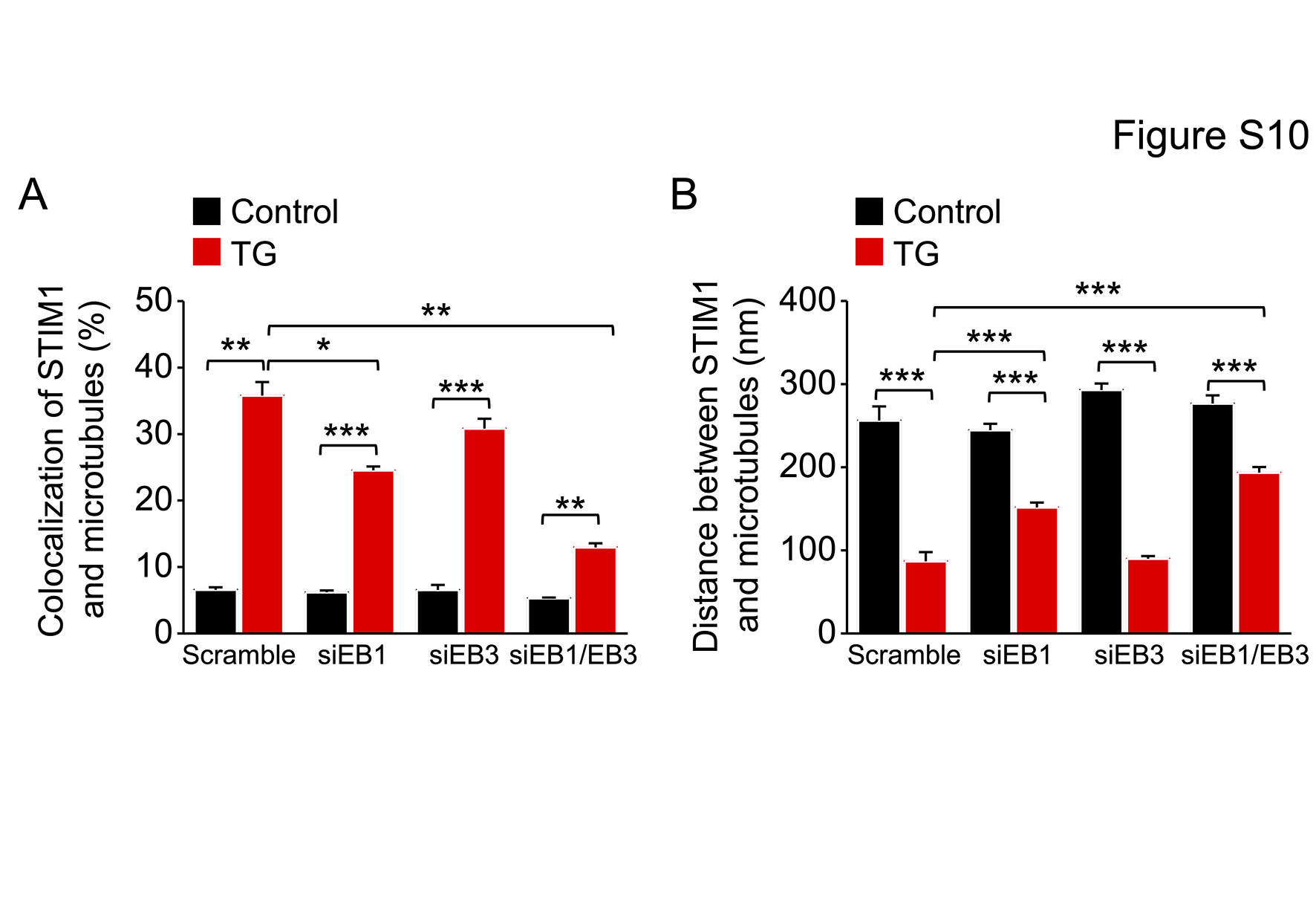

Supplement: Supplementary Figure 10 — The defect in interactions between STIM1 and microtubules after the downregulation of EB1. (A) The co-localization ratio of STIM1 and EB1 at the juxta-plasma membrane area was quantified by pixel-by-pixel analyses. Column, mean ± SEM from at least six different cells of three independent experiments. *P < 0.05, **P < 0.01, ***P < 0.001, compared with control group by one-way ANOVA with a Dunnett’s post hoc test. (B) Quantitative analyses of the molecular distances between STIM1 and microtubules with or without EB1 or EB3 silencing. Column, mean ± SEM from at least six different cells of three independent experiments. ***P < 0.001, compared with control group by one-way ANOVA with a Dunnett’s post hoc test. [file Image_10.TIFF]

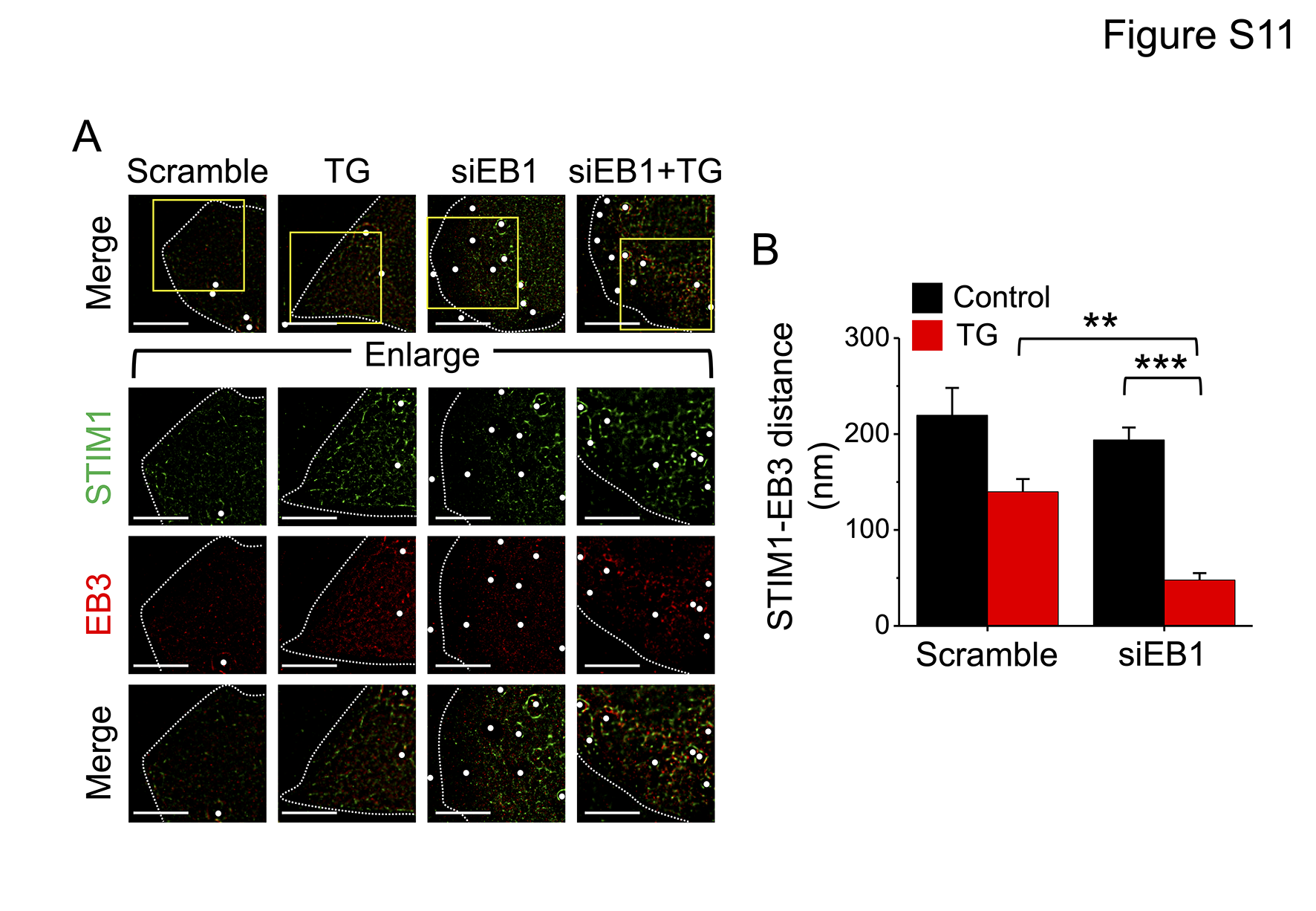

Supplement: Supplementary Figure 11 — Microtubule plus end-binding proteins play a compensatory role in regulating STIM1 trafficking. (A) Representative images showing that SiHa cells overexpressing EGFP-STIM1 were silenced for EB1 or not for 72 h. Lower panels showing the enlargement of the areas indicated by rectangles in whole-cell images. Dashed line, cell periphery. White dots, TetraSpeck microspheres. Scale bar, 10 μm. (B) Quantitative analyses of the molecular distances between STIM1 and EB3 with or without siEB1. Column, mean ± SEM from at least five different cells of three independent experiments. **P < 0.01, ***P < 0.001, compared with control group by one-way ANOVA with a Dunnett’s post hoc test. [file Image_11.TIFF]

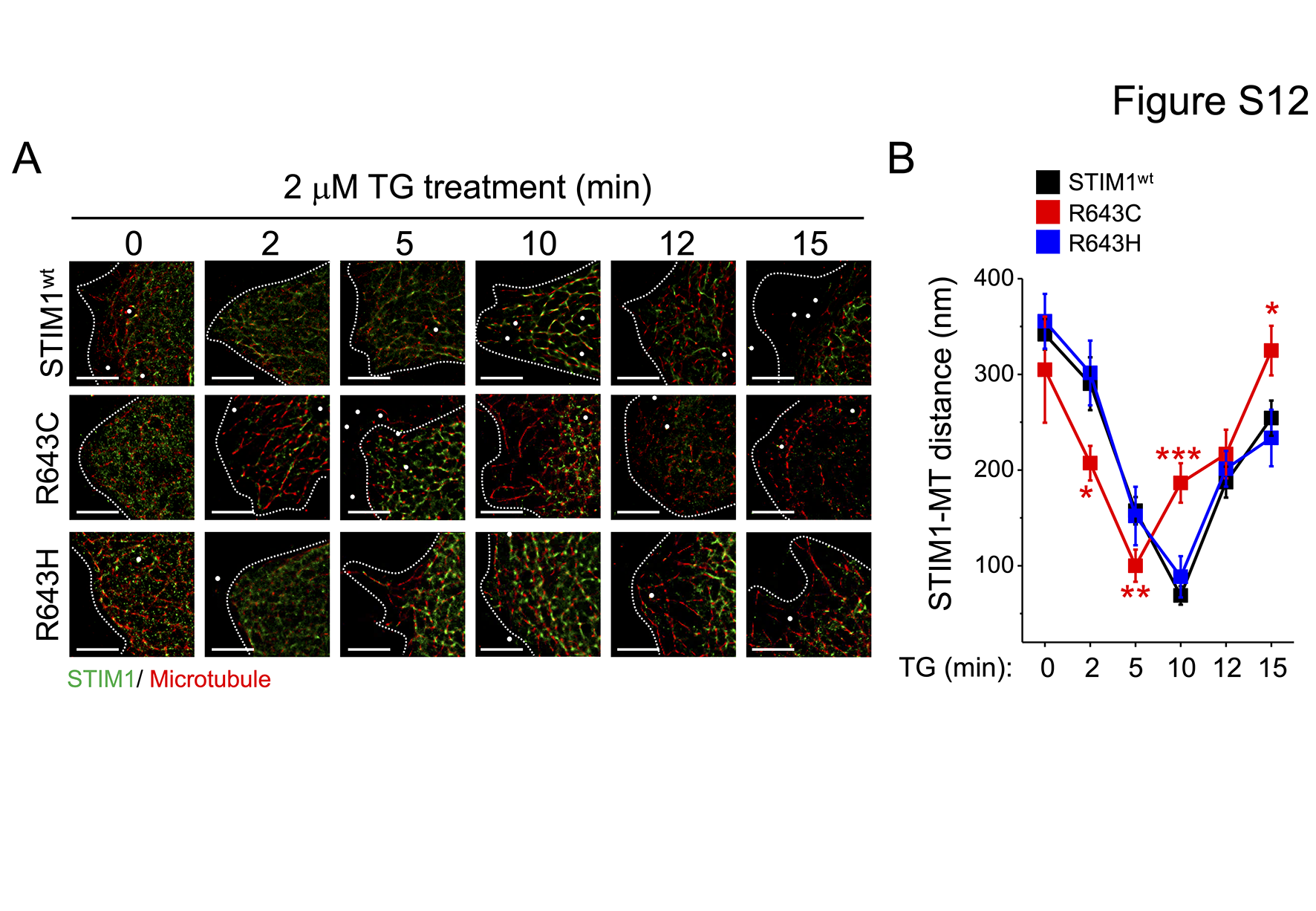

Supplement: Supplementary Figure 12 — Two mutations of STIM1 on S/TxIP motifs displayed distinguished cellular function. (A) Representative dSTORM images showing the intracellular distributions of SiHa cells with different STIM1 variants in TG-induced SOCE. Dashed line, cell periphery. White dots, TetraSpeck microspheres. Scale bar, 5 μm. (B) Quantitative analyses of the distances between SiHa cells with different STIM1 variants and microtubules. Each value represents the mean ± SEM from at least 30 ROIs of three independent experiments. *P < 0.05, **P < 0.01, ***P < 0.001, compared with wild-type group by one-way ANOVA with a Dunnett’s post hoc test. [file Image_12.TIFF]
