# Supplementary material for: Super-Resolution Microscopy Reveals That Stromal Interaction Molecule 1 Trafficking Depends on Microtubule Dynamics
Source: Front Physiol. 2021 Nov 5;12:762387. doi: 10.3389/fphys.2021.762387 (PMC8602801; doi:10.3389/fphys.2021.762387)
Supplement: Supplementary file 14 [file Data_Sheet_1.pdf]

Supplementary Table 1. The molecular distances between STIM1, microtubules, and EB1 during SOCE activation in nanoscale.

	**STIM1-MT**	**STIM1-EB1**	**MT-EB1**
**Control**	312.4 ± 17.3 nm	275.2 ± 18.2 nm	135.7 ± 10.2
**TG**	68.9 ± 9.5 nm	30.0 ± 4.3 nm	14.6 ± 4.8
***P* value**	<0.001	<0.001	<0.001

Summary of the quantitative analysis of molecular distances between STIM1, microtubules, and EB1 in TG-induced SOCE activation in nanoscale. P < 0.001, compared with control group.

Supplementary Table 2.

**Variants**	**Mutation CDS**	**Mutation frequency (% in tested samples)**	**Sample ID**	**Primary site**	**Primary histology**	**Histology subtype**
D76G	c.227A>G	0.00261978%	HCC08T	Liver	Carcinoma	Hepatocellular carcinoma
D76V	c.227A>T	0.00261978%	T3690	Large intestine	Carcinoma	Adenocarcinoma
D76H	c.226G>C	0.00261978%	RK190_C01	Liver	Carcinoma	*NS*
D84N	c.250G>A	0.00261978%	C91	Large intestine	Carcinoma	Intestinal adenocarcinoma
D84E	c.252T>A	0.00261978%	STC252	Stomach	Carcinoma	Adenocarcinoma
D84Y	c.250G>T	0.00261978%	YUMUL	Skin	Malignant melanoma	*NS*
R643C	c.1927C>T	0.00261978%	BK0097	Kidney	Carcinoma	Clear cell renal cell carcinoma
R643H	c.1928G>A	0.007859369%	TCGA-BR-8078-01	Stomach	Carcinoma	Adenocarcinoma
			sysucc-1397T	Large intestine	Carcinoma	Adenocarcinoma
			TCGA-AZ-6598-01	Large intestine	Carcinoma	Adenocarcinoma

The summary of the variants at STIM1 cEF hand and S/TxIP motif adapted from COSMIC database.

Supplementary Table 3

The antibodies used in this study.

Target	Catalog No.	Species	Source	Application	Dilution
GFP	GTX113617	Rabbit	GeneTex	Confocal; dSTORM	1:200
STIM1	610954	Mouse	BD	WB	1:5000
	ab108994	Rabbit	Abcam	Confocal; dSTORM	1:200; 1:200
STIM2	#4917	Rabbit	Cell Signaling	dSTORM	1:50
α-tubulin	NB100-690	Mouse	Novous	dSTORM	1:200
	ab52866	Rabbit	Abcam	WB; dSTORM	1:5000; 1:200
Ac-α-tubulin	T7451	Mouse	Sigma	dSTORM	1:200
EB1	sc-47704	Mouse	Santa Cruz	WB; dSTORM	1:3000; 1:50
	ab53358	Rat	Abcam	dSTORM	1:50
EB3	sc-136405	Mouse	Santa Cruz	WB; dSTORM	1:3000; 1:50
β-actin	GTX109639	Rabbit	GeneTex	WB	1:5000
	ab14128	Mouse	Abcam	WB	1:5000
Pyk2	#3292	Rabbit	Cell Signaling	WB	1:5000
p-Pyk2	#3291	Rabbit	Cell Signaling	WB; Confocal	1:3000; 1:200

WB, western blot; Confocal, FV-3000 Confocal Laser Scanning Microscope; dSTORM, dSTORM super-resolution microscopy. The dilutions used for each application are shown.

Supplementary Table 4

The primers for site directed mutagenesis of STIM1 variants.

STIM1 variants	Primer sequence (5'-3')
D76G	Forward: 5'-caccattggcatcatcgcccatcagtttgtggatg-3'
	Reverse: 5'-catccacaaactgatgggcgatgatgccaatggtg-3'
D76V	Forward: 5'-caccattggcatcatcgaccatcagtttgtggatg-3'
	Reverse: 5'-catccacaaactgatggtcgatgatgccaatggtg-3'
D76H	Forward: 5'-accattggcatcatcgtgcatcagtttgtggatgt-3'
	Reverse: 5'-acatccacaaactgatgcacgatgatgccaatggt-3'
D84Y	Forward: 5'-catcactttcttccacatacacatcaccattggcatc-3'
	Reverse: 5'-gatgccaatggtgatgtgtatgtggaagaaagtgatg-3'
D84E	Forward: 5'-tcatcactttcttccacttccacatcaccattggc-3'
	Reverse: 5'-gccaatggtgatgtggaagtggaagaaagtgatga-3'
D84N	Forward: 5'-catcactttcttccacattcacatcaccattggcatc-3'
	Reverse: 5'-gatgccaatggtgatgtgaatgtggaagaaagtgatg-3'
R643C	Forward: 5'-tggggaatgcatgtgtttcggctggcttgc-3'
	Reverse: 5'-gcaagccagccgaaacacatgcattcccca-3'
R643H	Forward: 5'-ccaggtggggaatgtgtgtgtttcggctg-3'
	Reverse: 5'-cagccgaaacacacacattccccacctgg-3'
